# Chemoenzymatic Synthesis of *ortho*-, *meta*-, and *para*-Substituted Derivatives of l-*threo*-3-Benzyloxyaspartate, An Important Glutamate Transporter Blocker

**DOI:** 10.1002/cctc.201500318

**Published:** 2015-06-16

**Authors:** Jandré de Villiers, Marianne de Villiers, Edzard M Geertsema, Hans Raj, Gerrit J Poelarends

**Affiliations:** Department of Pharmaceutical Biology, Groningen Research Institute of PharmacyUniversity of Groningen, Antonius Deusinglaan 1, 9713 AV Groningen (The Netherlands); Current address: Chr-Hansen A/S, Boge Alle 10-122970 Horsholm (Denmark)

**Keywords:** biocatalysis, excitatory amino acid transporters, glutamate, inhibitors, *threo*-3-benzyloxyaspartate

## Abstract

A simple, three-step chemoenzymatic synthesis of l-*threo*-3-benzyloxyaspartate (l-TBOA), as well as l-TBOA derivatives with F, CF_3_, and CH_3_ substituents at the aromatic ring, starting from dimethyl acetylenedicarboxylate was investigated. These chiral amino acids, which are extremely difficult to prepare by chemical synthesis, form an important class of inhibitors of excitatory amino acid transporters involved in the regulation of glutamatergic neurotransmission. In addition, a new chemical procedure for the synthesis of racemic mixtures of TBOA and its derivatives was explored. These chemically prepared racemates are valuable reference compounds in chiral-phase HPLC to establish the enantiopurities of the corresponding chemoenzymatically prepared amino acids.

l-Glutamate is the major excitatory neurotransmitter in the mammalian central nervous system and, as such, contributes to neuronal signaling through its activation of a variety of glutamate-gated ion channels.[1] Excitatory amino acid transporters (EAATs) are responsible for the uptake of glutamate from the synaptic cleft, which thereby terminates the glutamatergic neurotransmitter signal.[1,2] Hence, EAATs present on neurons and surrounding glia cells play a critical role in regulating synaptic glutamate concentrations. Accumulation of excitotoxic levels of extracellular glutamate may lead to overactivation of glutamate-gated ion channels and, consequently, neuronal injury. Glutamate-mediated neuronal injury has been linked to several neurological disorders such as amyotrophic lateral sclerosis, Alzheimer’s disease, epilepsy, and Huntington’s disease.[3]

Studies on EAATs, of which five subtypes (EAAT1–5) have been identified, have been largely dependent upon the development of selective and potent inhibitors that can be used to probe the physiological roles of these transporters in the regulation of glutamatergic neurotransmission or in the pathogenesis of neurological diseases.[1,3] Aspartate derivatives with large aryl or aryloxy substituents at the C3 position form an important class of inhibitors of EAATs.[3,4] This is exemplified by one of the first EAAT inhibitors to be reported, l-*threo*-3-benzyloxyaspartate (l-TBOA), which is a widely used nontransportable blocker for all five EAAT subtypes.[4a–c] However, the chemical synthesis of l-TBOA, the enantiomer of TBOA with the most potent inhibitory properties, is a highly challenging 11-step procedure starting from (*R*)-Garner aldehyde.[4c, d Therefore, there is great interest in the development of alternative procedures that provide simple and environmentally friendly access to l-TBOA and derivatives of l-TBOA with various substituents on the aromatic ring.

Several research groups have explored ammonia lyases as biocatalysts in the asymmetric amination of unsaturated acids to yield chiral α-amino acids.[5] This is a very attractive strategy for amino acid synthesis, because it makes use of readily available starting compounds without the requirement for recycling of cofactors, implementation of dynamic kinetic resolution strategies, or additional enzymes. The academic and industrial interest in aspartate derivatives, combined with the potential advantages of replacing chemical processes with biocatalysis, has prompted us to focus our attention on methylaspartate ammonia lyase (MAL).[6] This enzyme catalyzes the reversible addition of ammonia to mesaconate to give l-*threo*-3-methylaspartate and l-*erythro*-3-methylaspartate as products.[7] However, the wild-type enzyme has a narrow electrophile scope and only displays amination activity towards fumarate and its derivatives with a small substituent at the C2 position.[8]–[10] Interestingly, recent protein engineering work on MAL has shown that a glycine or an alanine mutation at position Leu384 resulted in mutant enzymes with the ability to aminate fumarate derivatives with large substituents at the C2 position.[10] The synthetic potential of the L384A mutant has been demonstrated by its use as a biocatalyst in the asymmetric synthesis of various 3-substituted aspartate derivatives, including the important EAAT inhibitor TBOA.[10,11]

In this report, we describe the synthetic potential of the L384A and L384G mutants of MAL in the selective preparation of both enantiomers of TBOA (**1 a**=l-TBOA), as well as a series of TBOA derivatives (i.e., **1 b**–**f**, **1 h**, and **1 i**) with different substituents at the *ortho*, *meta*, and *para* positions of the aromatic ring (Scheme 1). This convenient three-step chemoenzymatic method for the stereoselective synthesis of TBOA and ring-substituted derivatives of TBOA (starting from dimethyl acetylenedicarboxylate **4**) appears to be an elegant alternative for existing, highly challenging multistep-synthesis procedures.[[Bibr b4c][Bibr b4d]]

**Scheme 1:**
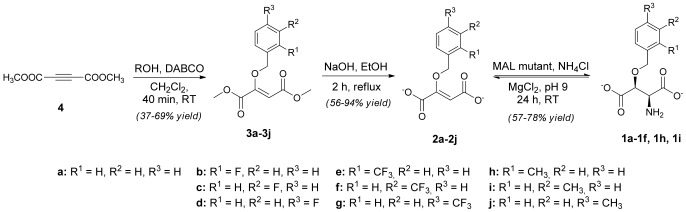
Chemoenzymatic synthesis of TBOA and its derivatives by using either the L384G or L384A mutant of MAL as biocatalyst. DABCO=1,4-diazabicyclo[2.2.2]octane.

To investigate the efficiency of the L384G and L384A mutants to aminate 2-benzyloxyfumarate (**2 a**), kinetic parameters were determined (Table 1). The data indicate that the two MAL mutants aminate **2 a** with similar kinetic parameters and catalytic efficiency, having respectable *k*_cat_ values of 6–7 s^−1^. We also compared the ability of these mutant enzymes to aminate **2 a** under optimized reaction conditions (Figure 1). With 0.01 mol % biocatalyst and a 100-fold molar excess of ammonia (5 M) relative to the amount of **2 a** (50 mM), the reactions were complete within just a few hours at pH 9.0 and room temperature, and excellent conversions of >80 % were achieved. These results clearly demonstrate the potential of these mutants for application in the synthesis of 3-benzyloxyaspartate.

**Table 1:** Apparent kinetic parameters for the MAL(L384A)- and MAL(L384G)-catalyzed addition of ammonia to 2-benzyloxyfumarate (2 a).[a]

MAL mutant	*k*_cat_ [s^−1^]	*K*_m_ (for2 a) [mM]	*K*_m_ (for NH_3_) [mM]	*k*_cat_/*K*_m_ (for2 a) [M^−1^ s^−1^]
L384A	6.8±0.3	18±1.9	438±66	378
L384G	6.5±0.2	12±1	410±56	542

[a] [a] Each experiment was done in triplicate. Errors are standard deviations from each fit. *k*_cat_=turnover number, *K*_m_=Michaelis constant.

**Figure 1:**
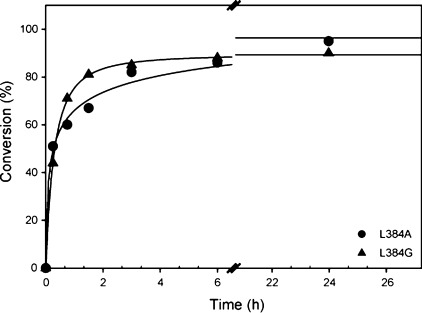
Enzyme-catalyzed transformations under optimized reaction conditions. Progress curves for the L384A- and L384G-catalyzed (0.01 mol %) addition of ammonia (5 M) to 2-benzyloxyfumarate (2a, 50 mM), as monitored by ^1^H NMR spectroscopy.

To further demonstrate the synthetic usefulness of the MAL mutants, a preparative-scale reaction using 0.01 mol % biocatalyst (the L384G mutant), ammonia (5 M), and **2 a** (0.75 mmol, 50 mM) was performed at pH 9.0 and room temperature. The reaction was stopped after 24 h at a final conversion of 90 % (Table 2, entry 1). The amino acid product was purified (78 % yield) and identified as the desired *threo* isomer of 3-benzyloxyaspartate (*de*>95 %) by comparison of its ^1^H NMR signals to those of chemically synthesized authentic standards with known *threo* or *erythro* configuration (see the Supporting Information). No other regio- or diastereoisomer was observed. The absolute configuration of the product was determined by high-performance liquid chromatography (HPLC) on a chiral stationary phase by using chemically prepared authentic standards with known dl-*threo* or l-*threo* configuration (Figure S3, Supporting Information). This analysis revealed that the product had the l-*threo* configuration and was present as a single enantiomer (**1 a**, l-TBOA; *ee*>99 %).

**Table 2:** MAL(L384A)- and MAL(L384G)-catalyzed addition of ammonia to fumarate derivatives 2 a–j.

Entry	Fumarate derivative	Product	Catalyst [mol %]	L384A conversion [%]	L384G conversion [%]	Yield [%][a]	*de* [%][b]	*ee* [%]		
				3 h	24 h	3 h	24 h			
1	**2 a**	**1 a**	0.01	88	95	83	90	78	>95[c]	>99[e]
2	**2 b**	**1 b**	0.01	54	91	45	89	60	>95[d]	ND[f]
3	**2 c**	**1 c**	0.01	95	95	93	94	61	>95[d]	>99[e]
4	**2 d**	**1 d**	0.01	62	94	88	92	57	>95[d]	ND[f]
5	**2 e**	**1 e**	0.05	61	95	50	92	58	>95[d]	ND[f]
6	**2 f**	**1 f**	0.05	0	0	92	94	77	>95[d]	>99[e]
7	**2 g**	–	0.05	0	0	0	0	–	–	–
8	**2 h**	**1 h**	0.05	91	92	86	90	71	>95[d]	ND[f]
9	**2 i**	**1 i**	0.05	91	93	92	95	73	>95[c]	>99[e]
10	**2 j**	–	0.05	0	0	0	0	–	–	–

[a] Yield of isolated product after ion-exchange chromatography.

[b] The diastereomeric excess (*de*) is defined as the excess of *threo* isomer over the *erythro* isomer.

[c] The purified amino acid product had the *threo* configuration, as determined by comparison of its ^1^H NMR signals to those of chemically synthesized authentic standards with known *threo* or *erythro* configuration.

[d] The purified amino acid product was tentatively assigned the *threo* configuration on the basis of analogy (see the Supporting Information).

[e] The enantiomeric excess of the isolated product was determined by HPLC on a chiral stationary phase by using a chemically synthesized authentic standard with known dl-*threo* configuration.

[f] ND=not determined; however, the optical rotations of products **1 b**, **1 d**, **1 e**, and **1 h** (in terms of both signs and values) are identical to those of enantiopure products **1 a**, **1 c**, **1 f**, and **1 i** (Table S1), which may suggest high optical purity for products **1 b**, **1 d**, **1 e**, and **1 h**.

The preparative-scale experiment was repeated under identical conditions but by using the L384A mutant as a biocatalyst instead of the L384G mutant. Analysis of the isolated amino acid product revealed that the L384A-catalyzed amination of **2 a** was also highly regio- and stereoselective and that l-TBOA was exclusively produced (*de*>95 %, *ee*>99 %). We also explored the L384A mutant for the kinetic resolution of a racemic mixture of dl-TBOA (5 mg, 0.021 mmol) in an analytical-scale experiment. The reaction was followed by monitoring substrate depletion and product formation by HPLC on a chiral stationary phase. With 0.1 mol % biocatalyst, the reaction was complete within 24 h at pH 8.0 and room temperature, and near 50 % conversion of the starting material was achieved (Figure S9). The remaining amino acid product, D-TBOA, had an *ee* value of >95 %. Taken together, the results clearly demonstrate the potential of the L384A and L384G mutants for the selective preparation of both enantiomers of TBOA.

Next, a series of 2-benzyloxyfumarate derivatives **2 b**–**j** (Scheme 1) was prepared from dimethyl acetylenedicarboxylate (**4**) and evaluated as substrates for the L384G and L384A mutants. The ability of these MAL mutants to catalyze the amination of substrates with a small (e.g., F) or more bulky (e.g., CF_3_ and CH_3_) substituent at the *ortho*, *meta*, or *para* position of the aromatic ring (i.e., **2 b**–**j**) was tested by using ^1^H NMR spectroscopy (Table 2). The L384A mutant displayed activity for 2-benzyloxyfumarate derivatives **2 b**–**e**, **2 h**, and **2 i**. Reaction mixtures were incubated at pH 9.0 and room temperature, and conversions of >50 % after 3 h and >90 % after 24 h were achieved. Strikingly, mutant L384G not only showed activity for compounds **2 b**–**e**, **2 h**, and **2 i**, but it also processed 2-benzyloxyfumarate derivative **2 f**, which was not converted at all by the L384A mutant. Again, excellent conversions were achieved after incubation for 24 h at pH 9.0 and room temperature. The aryl binding pocket of the L384G mutant is most likely slightly larger than that of the L384A mutant,[10] and this rationalizes its ability to convert compound **2 f**, which carries a large trifluoromethyl group at the *meta* position. Compounds with a trifluoromethyl (**2 g**) or methyl (**2 j**) substituent at the *para* position were not accepted as substrates by the L384A mutant nor the L384G mutant. Because the L384G mutant showed a larger substrate scope, it was selected as the biocatalyst for application in preparative-scale reactions.

Preparative-scale experiments were performed to allow unambiguous product identification by HRMS and ^1^H NMR, ^13^C NMR, and/or ^19^F NMR spectroscopy and thus to ascertain that the L384G-catalyzed amination of **2 b**–**f**, **2 h**, and **2 i** yields the corresponding amino acid products **1 b**–**f**, **1 h**, and **1 i** (Scheme 1). Ammonia (5 M), 2-benzyloxyfumarate derivative (0.75 mmol, 50 mM), and biocatalyst (either 0.01 or 0.05 mol %) were incubated at pH 9.0 and room temperature, and reactions were stopped after 24 h, which resulted in excellent conversions of >89 % (Table 2). The products were purified (57–77 % yield) and identified as the corresponding 3-benzyloxyaspartate derivatives **1 b**–**f**, **1 h**, and **1 i**. Amino acid product **1 i** was further identified as the desired *threo* isomer of 3-(3-methyl)benzyloxyaspartate (*de*>95 %) by comparison of its ^1^H NMR signals to those of chemically synthesized authentic standards with known *threo* or *erythro* configuration. Although the relative configuration of products **1 b**–**f** and **1 h** was not determined by comparison to authentic standards, we assume the relative configuration to be *threo* for all products on the basis of analogy (see the Supporting Information).[10] Analysis of products **1 c**, **1 f**, and **1 i** by HPLC on a chiral stationary phase by using chemically prepared authentic standards with known dl-*threo* configuration revealed that the L384G-catalyzed amination reactions were highly enantioselective and that these amino acids were produced with >99 % *ee* (Table 2; Figures S5–S7). Control experiments showed that the 2-benzyloxyfumarate derivatives did not undergo amination in the absence of the enzyme. These results show that the L384G mutant has striking potential for application in the regio- and stereoselective synthesis of derivatives of TBOA.

In conclusion, we demonstrated the chemoenzymatic synthesis of l-*threo*-3-benzyloxyaspartate (l-TBOA) and various derivatives of TBOA with substituents on the aromatic ring by asymmetric amination of 2-benzyloxyfumarate derivatives by using engineered methylaspartate ammonia lyase variants. This chemoenzymatic approach appears to be an elegant alternative for existing chemical methods.[4a–d] This was convincingly demonstrated by the 3-step chemoenzymatic synthesis of l-TBOA, the selective chemical preparation of which is a highly challenging 11-step procedure starting from (*R*)-Garner aldehyde.[4c] Appropriate experiments to investigate the inhibitory properties of the newly synthesized TBOA derivatives against aspartate/glutamate transporters are underway.
