# Supplementary material for: Chemoenzymatic Synthesis of ortho-, meta-, and para-Substituted Derivatives of l-threo-3-Benzyloxyaspartate, An Important Glutamate Transporter Blocker
Source: ChemCatChem. 2015 Jun 16;7(13):1931–4. doi: 10.1002/cctc.201500318 (PMC4517298; doi:10.1002/cctc.201500318)
Supplement: Supplementary file 1 [file cctc0007-1931-sd1.pdf]

Heterogeneous & Homogeneous & Bio-

CHEMCATCHEM

CATALYSIS

Supporting Information

Chemoenzymatic Synthesis of *ortho*-, *meta*-, and *para*-Substituted Derivatives of L-*threo*-3-Benzyloxyaspartate, An Important Glutamate Transporter Blocker

Jandré de Villiers,[a] Marianne de Villiers,[a] Edzard M. Geertsema,[a] Hans Raj,[a, b] and Gerrit J. Poelarends*[a]

cctc_201500318_sm_miscellaneous_information.pdf

EXPERIMENTAL PROCEDURES

Materials and general methods

All chemicals were purchased from Sigma-Aldrich Chemical Co. (St. Louis, MO) unless stated otherwise. Solvents were purchased from Biosolve (Valkenswaard, The Netherlands) or Sigma-Aldrich Chemical Co. Ingredients for buffers and media were obtained from Duchefa Biochemie (Haarlem, The Netherlands) or Merck (Darmstadt, Germany). Ni-Sepharose 6 fast flow resin and prepacked PD-10 Sephadex G-25 columns for protein purification were purchased from GE Healthcare Bio-Sciences (Little Chalfont, UK). Supelco C18 SPE prepacked cartridges were bought from Sigma-Aldrich Chemical Co. Proteins were analyzed by sodium dodecyl sulfate polyacrylamide gel electrophoresis (SDS-PAGE) under denaturing conditions on gels containing 10% polyacrylamide. The gels were stained with Coomassie brilliant blue. Kinetic data were obtained on a V-660 spectrophotometer from Jasco (IJsselstein, The Netherlands). High performance liquid chromatography (HPLC) was performed with a Shimadzu LC-10AT HPLC with a Shimadzu SPD-M10A photodiode array detector. NMR analyses were performed on a Varian Inova 200, 300, 400 or 500 MHz machine at the NMR Center, University of Groningen, or on a Brucker 500 MHz machine at the Drug Design laboratory of the University of Groningen. Chemical shifts (δ) are reported in parts per million (ppm). Electrospray ionization mass spectrometry (ESI-MS) and orbitrap HRMS were performed by the Mass Spectrometry core facility, University of Groningen.

Synthesis of dimethyl 2-benzyloxyfumarate and its derivatives (3a-3j)

The synthesis of the dimethyl 2-benzyloxyfumarate derivatives **3b-3j** was based on a previously published procedure for dimethyl 2-benzyloxyfumarate (**3a**).[1] Briefly, to a solution of dimethyl acetylenedicarboxylate (0.711 g, 5 mmol) in dichloromethane (40 mL) was added DABCO (0.0561 g, 0.5 mmol) followed by the appropriate alcohol (5 mmol). The reaction was stirred at room temperature for 40 min or until all the starting material was consumed (TLC, EtOAc:Heptane 1:4). The solvent was removed under reduced pressure to give the crude product as a dark purple/brown oil. The *E/Z* isomers of the products were separated by flash chromatography (silica gel, EtOAc:Heptane). The preferred *E* isomer (i.e., the fumarate derivative) was used in further experiments.

Dimethyl 2-benzyloxyfumarate (3a)

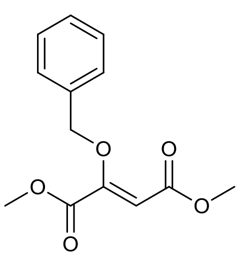

White solid. 863 mg (69% yield). ^{1}H NMR ($CDCl_3$, 300 MHz, 25°C): δ = 3.74 (s, 3H, COOC**H**$_3$), 3.82 (s, 3H, COOC**H**$_3$), 5.19 (s, 2H, OC**H**$_2$Ph), 6.25 (s, 1H, C=C**H**), 7.35–7.37 (m, 5H, Ar). ^{1}H NMR signals are in agreement with the literature data.[1]

Dimethyl 2-(2-fluoro)benzyloxyfumarate (3b)

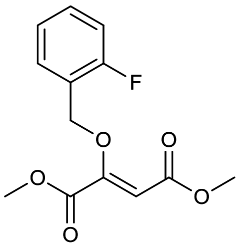

White solid. 657 mg (49% yield). ^{1}H NMR ($CDCl_3$, 300 MHz, 25°C): δ = 3.72 (s, 3H, COOC**H**$_3$), 3.84 (s, 3H, COOC**H**$_3$), 5.25 (s, 2H, OC**H**$_2$Ph), 6.29 (s, 1H, C=C**H**), 7.01–7.20 (m, 2H, Ar), 7.27–7.34 (m, 1H, Ar), 7.56 (td, *J* = 7.4; 1.8 Hz, 1H, Ar); ^{13}C NMR ($CDCl_3$, 75.5 MHz, 25°C): δ = 52.0, 53.1,

69.0, 111.1, 115.5 (d, J = 21.3 Hz), 124.4 (d, J = 3.7 Hz), 130.4 (d, J = 8.2 Hz), 130.6 (d, J = 24.1 Hz), 130.9 (d, J = 3.6 Hz), 153.7, 160.9 (d, J = 255.5 Hz), 163.3, 164.7; ^{19}F NMR ($CDCl_3$, 200 MHz, 25°C): δ = –118.2; MS (ESI): m/z (%) = 269.0 (100) $[M+H]^+$.

Dimethyl 2-(3-fluoro)benzyloxyfumarate (3c)

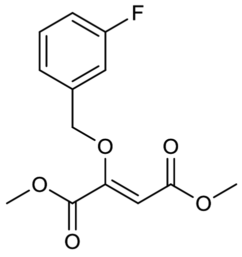

White solid. 604 mg (45% yield). ^{1}H NMR ($CDCl_3$, 300 MHz, 25°C): δ = 3.75 (s, 3H, COOC**H**$_3$), 3.84 (s, 3H, COOC**H**$_3$), 5.18 (s, 2H, OC**H**$_2$Ph), 6.29 (s, 1H, C=C**H**), 6.97–7.06 (m, 1H, Ar), 7.19–7.21 (m, 2H, Ar), 7.28–7.34 (m, 1H, Ar); ^{13}C NMR ($CDCl_3$, 75.5 MHz, 25°C): δ = 51.9, 53.1, 74.3, 110.8, 115.0 (d, J = 22.1 Hz), 115.3 (d, J = 21.2 Hz), 123.5 (d, J = 2.9 Hz), 130.1 (d, J = 8.2 Hz), 138.8 (d, J = 7.4 Hz), 153.5, 162.8 (d, J = 222.0 Hz), 164.7, 165.4; ^{19}F NMR ($CDCl_3$, 200 MHz, 25°C): δ = –112.9; MS (ESI): m/z (%) = 269.3 (100) $[M+H]^+$.

Dimethyl 2-(4-fluoro)benzyloxyfumarate (3d)

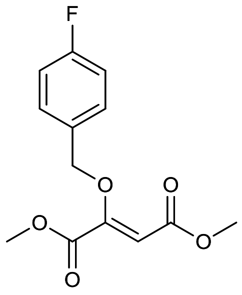

White solid. 644 mg (48% yield). ^{1}H NMR ($CDCl_3$, 300 MHz, 25°C): δ = 3.74 (s, 3H, COOC**H**$_3$), 3.83 (s, 3H, COOC**H**$_3$), 5.15 (s, 2H, OC**H**$_2$Ph), 6.28 (s, 1H, C=C**H**), 7.00–7.08 (m, 2H, Ar), 7.31–7.45 (m, 2H, Ar); ^{13}C NMR ($CDCl_3$, 75.5 MHz, 25°C): δ = 51.9, 53.0, 74.4, 110.8, 115.5 (d, J = 21.6 Hz), 130.3 (d, J = 8.3 Hz), 132.0 (d, J = 3.4 Hz), 153.6, 162.5 (d, J = 213.0 Hz), 163.4, 165.3; ^{19}F NMR ($CDCl_3$, 200 MHz, 25°C): δ = –113.6; MS (ESI): m/z (%) = 269.2 (100) $[M+H]^+$.

Dimethyl 2-(2-trifluoromethyl)benzyloxyfumarate (3e)

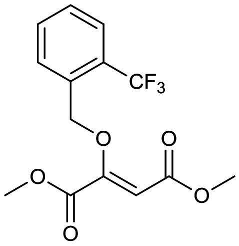

White solid. 720 mg (45% yield). ^{1}H NMR ($CDCl_3$, 300 MHz, 25°C): δ = 3.74 (s, 3H, COOC**H**$_3$), 3.85 (s, 3H, COOC**H**$_3$), 5.35 (s, 2H, OC**H**$_2$Ph), 6.34 (s, 1H, C=C**H**), 7.42 (t, J = 7.5 Hz, 1H, Ar), 7.62 (dd, J = 15.4; 7.7 Hz, 2H, Ar), 7.97 (d, J = 7.7 Hz, 1H, Ar); ^{13}C NMR ($CDCl_3$, 75.5 MHz, 25°C): δ = 52.0, 53.1, 71.0, 110.8, 124.4 (q, J = 273.5 Hz), 125.8 (q, J = 5.7 Hz), 127.3 (q, J = 30.8 Hz), 128.0, 129.5, 132.3 (q, J = 1.1 Hz), 135.1, 153.6, 163.3, 164.7; ^{19}F NMR ($CDCl_3$, 200 MHz, 25°C): δ = –60.1; MS (ESI): m/z (%) = 319.0 (100) $[M+H]^+$.

Dimethyl 2-(3-trifluoromethyl)benzyloxyfumarate (3f)

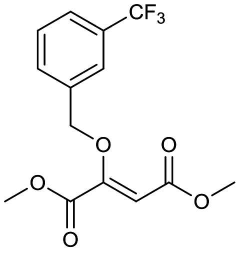

White solid. 710 mg (45% yield). ^{1}H NMR ($CDCl_3$, 300 MHz, 25°C): δ = 3.76 (s, 3H, COOC**H**$_3$), 3.85 (s, 3H, COOC**H**$_3$), 5.23 (s, 2H, OC**H**$_2$Ph), 6.32 (s, 1H, C=C**H**), 7.49 (t, J = 7.7 Hz, 1H, Ar), 7.59 (d, J = 7.7 Hz, 1H, Ar), 7.66 (d, J = 7.6 Hz, 1H, Ar), 7.74 (s, 1H, Ar); ^{13}C NMR ($CDCl_3$, 75.5 MHz, 25°C): δ = 52.0, 53.1, 74.3, 111.3, 124.9 (q, J = 3.8 Hz), 125.2 (q, J = 3.9 Hz), 129.1, 131.3, 137.3, 153.4, 163.3, 164.6 (two signals not visible due to low resolution of spectrum: $\underline{C}F_3$ and $\underline{C}CF_3$); ^{19}F NMR ($CDCl_3$, 200 MHz, 25°C): δ = –62.7; MS (ESI): m/z (%) = 336.1 (100) $[M+H_2O]^+$.

Dimethyl 2-(4-trifluoromethyl)benzyloxyfumarate (3g)

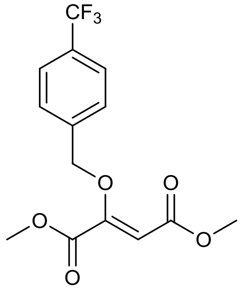

White solid. 588 mg (37% yield). ^{1}H NMR ($CDCl_3$, 300 MHz, 25°C): δ = 3.75 (s, 3H, COOC**H**$_3$), 3.85 (s, 3H, COOC**H**$_3$), 5.24 (s, 2H, OC**H**$_2$Ph), 6.33 (s, 1H, C=C**H**), 7.61 (q, J = 8.4 Hz, 4H, Ar); ^{13}C NMR ($CDCl_3$, 75.5 MHz, 25°C): δ = 52.0, 53.1, 74.2, 110.9, 124.1 (q, J = 266.9 Hz), 125.6 (q, J = 3.8 Hz), 128.1, 153.5, 163.3, 164.6 (two signals not visible due to low resolution of spectrum: <u>C</u>CH$_2$O and <u>C</u>CF$_3$); ^{19}F NMR ($CDCl_3$, 200 MHz, 25°C): δ = –62.7; MS (ESI): m/z (%) = 319.0 (75) $[M+H]^+$.

Dimethyl 2-(2-methyl)benzyloxyfumarate (3h)

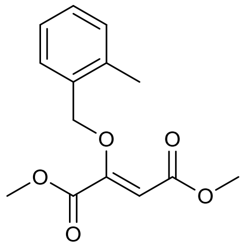

White solid. 660 mg (50% yield). ^{1}H NMR ($CDCl_3$, 300 MHz, 25°C): δ = 2.41 (s, 3H, Ar–C**H**$_3$), 3.73 (s, 3H, COOC**H**$_3$), 3.81 (s, 3H, COOC**H**$_3$), 5.19 (s, 2H, OC**H**$_2$Ph), 6.24 (s, 1H, C=C**H**), 7.17–7.23 (m, 3H, Ar), 7.40 (d, J = 6.8 Hz, 1H, Ar); ^{13}C NMR ($CDCl_3$, 75.5 MHz, 25°C): δ = 19.0, 51.9, 53.0, 73.6, 110.1, 126.1, 128.9, 129.7, 130.6, 137.5, 147.5, 153.9, 163.7, 164.9; MS (ESI): m/z (%) = 265.1 (95) $[M+H]^+$.

Dimethyl 2-(3-methyl)benzyloxyfumarate (3i)

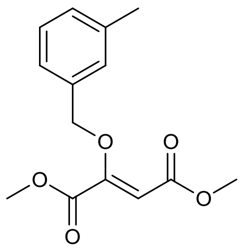

White solid. 770 mg (58% yield). ^{1}H NMR ($CDCl_3$, 300 MHz, 25°C): δ = 2.36 (s, 3H, Ar–C**H**$_3$), 3.74 (s, 3H, COOC**H**$_3$), 3.83 (s, 3H, COOC**H**$_3$), 5.15 (s, 2H, OC**H**$_2$Ph), 6.25 (s, 1H, C=C**H**), 7.13–7.26 (m, 4H, Ar); ^{13}C NMR

(CDCl$_3$, 75.5 MHz, 25°C): δ = 21.6, 51.9, 53.1, 75.4, 110.5, 125.6, 128.6, 129.2, 129.3, 136.2, 138.3, 153.8, 163.6, 164.9; MS (ESI): *m/z* (%) = 287.1 (100) $[M+Na]^+$.

Dimethyl 2-(4-methyl)benzyloxyfumarate (3j)

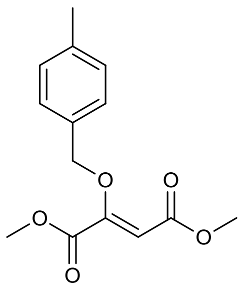

White solid. 710 mg (53% yield). ^{1}H NMR (CDCl$_3$, 300 MHz, 25°C): δ = 2.34 (s, 3H, Ar–C**H**$_3$), 3.74 (s, 3H, COOC**H**$_3$), 3.82 (s, 3H, COOC**H**$_3$), 5.16 (s, 2H, OC**H**$_2$Ph), 6.23 (s, 1H, C=C**H**), 7.17 (d, *J* = 7.8 Hz, 2H, Ar), 7.31 (d, *J* = 7.9 Hz, 2H, Ar); ^{13}C NMR (CDCl$_3$, 75.5 MHz, 25°C): δ = 21.4, 51.8, 53.0, 75.1, 110.3, 128.6, 129.3, 133.1, 138.4, 153.8, 163.6, 165.8; MS (ESI): *m/z* (%) = 265.0 (30) $[M+H]^+$.

Synthesis of 2-benzyloxyfumaric acid and its derivatives (2a-2j)

The synthesis of **2a-2j** was based on a procedure published elsewhere.[2] Briefly, the appropriate dimethyl 2-benzyloxyfumarate derivative (3 mmol) was dissolved in EtOH (6 mL). To this mixture was added 2 M NaOH (6 mL) after which the reaction mixture was refluxed for 2 h. The mixture was allowed to cool to room temperature and washed with EtOAc (1 x 10 mL). The aqueous layer was acidified to pH 1 with a conc. HCl solution followed by extraction with EtOAc (4 x 10 mL). The combined organic layers were dried over MgSO$_4$, filtered and the solvent removed *in vacuo*. The resulting white solid was precipitated from Et$_2$O: pentane to give the final product as a white solid.

2-benzyloxyfumaric acid (2a)

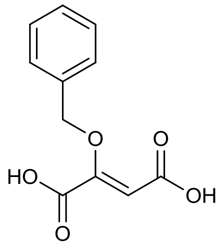

White solid. 373 mg (56% yield). Mp 132–133°C (neat); ^{1}H NMR (DMSO–d6, 300 MHz, 25°C): δ = 5.10 (s, 2H, OC**H**$_2$Ph), 6.07 (s, 1H, C=C**H**), 7.32–7.43 (m, 5H, Ar); ^{13}C NMR (DMSO–d6, 75.5 MHz, 25°C): δ = 73.6, 110.3, 127.8, 128.0, 128.3, 136.6, 153.6, 164.0, 165.3; HRMS–ESI: *m/z* [M]$^-$ calcd for $C_{11}H_9O_5$: 221.04555; found: 221.04571. ^{1}H NMR signals are in agreement with the literature data.[2]

2-(2-fluoro)benzyloxyfumaric acid (2b)

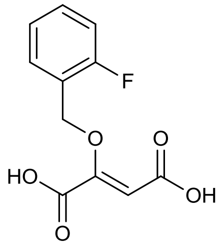

White solid. 570 mg (79% yield). Mp 129–130°C (diethyl ether); ^{1}H NMR (DMSO–d6, 300 MHz, 25°C): δ = 5.18 (s, 2H, OC**H**$_2$Ph), 6.10 (s, 1H, C=C**H**), 7.18–7.25 (m, 2H, Ar), 7.35–7.45 (m, 1H, Ar), 7.51–7.67 (m, 1H, Ar); ^{13}C NMR (DMSO–d6, 75.5 MHz, 25°C): δ = 67.5 (d, *J* = 4.1 Hz), 110.5, 115.2 (d, *J* = 21.1 Hz), 123.5 (d, *J* = 14.3 Hz), 124.4 (d, *J* = 3.5 Hz), 130.0 (d, *J* = 21.7 Hz), 130.6 (d, *J* = 9.4 Hz), 153.2, 160.1 (d, *J* = 246.3 Hz), 163.9, 165.2; ^{19}F NMR (DMSO–d6, 200 MHz, 25°C): δ = –118.4; HRMS–ESI: *m/z* [M]$^-$ calcd for $C_{11}H_8FO_5$: 239.03613; found: 239.03627.

2-(3-fluoro)benzyloxyfumaric acid (2c)

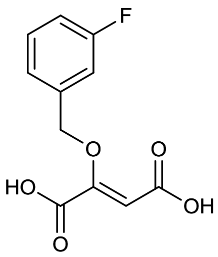

White solid. 613 mg (85% yield). Mp 139–140°C (diethyl ether); ^{1}H NMR (DMSO–d6, 300 MHz, 25°C): δ = 5.13 (s, 2H, OC**H**$_2$Ph), 6.11 (s, 1H, C=C**H**), 7.11–7.18 (m, 1H, Ar), 7.23–7.32 (m, 2H, Ar), 7.36–7.46 (m, 1H, Ar); ^{13}C NMR (DMSO–d6, 75.5 MHz, 25°C): δ = 72.8 (d, J = 1.8 Hz), 110.7, 114.3 (d, J = 21.9 Hz), 114.7 (d, J = 20.7 Hz), 123.5 (d, J = 2.8 Hz), 130.3 (d, J = 8.3 Hz), 139.7 (d, J = 7.5 Hz), 153.5, 162.1 (d, J = 243.3 Hz), 164.0, 165.3; ^{19}F NMR (DMSO–d6, 200 MHz, 25°C): δ = –113.3; HRMS–ESI: *m/z* [M]$^-$ calcd for $C_{11}H_8FO_5$: 239.03613; found: 239.0363.

2-(4-fluoro)benzyloxyfumaric acid (2d)

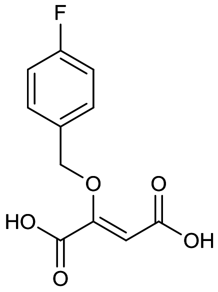

White solid. 454 mg (63% yield). Mp 132–133°C (diethyl ether); ^{1}H NMR (DMSO–d6, 400 MHz, 25°C): δ = 5.08 (s, 2H, OC**H**$_2$Ph), 6.09 (s, 1H, C=C**H**), 7.16–7.23 (m, 2H, Ar), 7.43–7.50 (m, 2H, Ar); ^{13}C NMR (DMSO–d6, 75.5 MHz, 25°C): δ = 72.9, 110.6, 115.0 (d, J = 21.5 Hz), 130.1 (d, J = 8.4 Hz), 132.8 (d, J = 3.0 Hz), 153.4, 161.8 (d, J = 243.8 Hz), 164.0, 165.2; ^{19}F NMR (DMSO–d6, 200 MHz, 25°C): δ = –112.1; HRMS–ESI: *m/z* [M]$^-$ calcd for $C_{11}H_8FO_5$: 239.03613; found: 239.03629.

2-(2-trifluoromethyl)benzyloxyfumaric acid (2e)

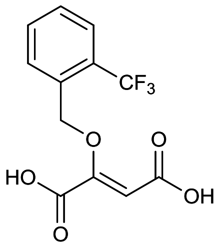

White solid. 504 mg (58% yield). Mp 131–132°C (diethyl ether); ^{1}H NMR (DMSO–d6, 300 MHz, 25°C): δ = 5.30 (s, 2H, OC**H**$_2$Ph), 6.16 (s, 1H, C=C**H**), 7.55 (t, J = 7.6 Hz, 1H, Ar), 7.73 (t, J = 7.7 Hz, 2H, Ar), 7.94 (d, J = 7.6 Hz, 1H, Ar); ^{13}C NMR (DMSO–d6, 75.5 MHz, 25°C): δ = 70.1, 110.8, 124.6 (q, J = 273.6 Hz), 126.0 (q, J = 5.6 Hz), 127.0 (q, J = 35.9 Hz), 128.9, 130.1, 133.1 (q, J = 1.3 Hz), 135.4 (q, J = 1.6 Hz), 153.5, 164.2, 165.7; ^{19}F NMR (DMSO–d6, 200 MHz, 25°C): δ = –58.6; HRMS–ESI: *m/z* [M]$^-$ calcd for $C_{12}H_8F_3O_5$: 289.03293; found: 289.03311.

2-(3-trifluoromethyl)benzyloxyfumaric acid (2f)

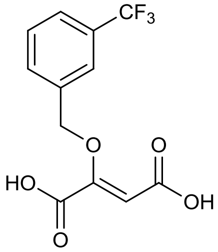

White solid. 618 mg (71% yield). Mp 144–145°C (diethyl ether); ^{1}H NMR (DMSO–d6, 300 MHz, 25°C): δ = 5.13 (s, 2H, OC**H**$_2$Ph), 6.10 (s, 1H, C=C**H**), 7.16 (t, J = 8.6 Hz, 1H, Ar), 7.22–7.34 (m, 2H, Ar), 7.36–7.41 (m, 1H, Ar); ^{13}C NMR (DMSO–d6, 75.5 MHz, 25°C): δ = 73.2, 111.4, 124.6 (q, J = 3.8 Hz), 124.7 (q, J = 272.2 Hz), 125.0 (q, J = 3.8 Hz), 129.5 (q, J = 31.8 Hz), 129.8, 131.9, 138.7, 154.0, 164.4, 165.6; ^{19}F NMR (DMSO–d6, 200 MHz, 25°C): δ = –61.1; HRMS–ESI: *m/z* [M]$^-$ calcd for $C_{12}H_8F_3O_5$: 289.03293; found: 289.03314.

2-(4-trifluoromethyl)benzyloxyfumaric acid (2g)

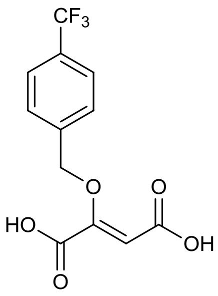

White solid. 601 mg (69% yield). Mp 154–155°C (diethyl ether); ^{1}H NMR (DMSO–d6, 300 MHz, 25°C): δ = 5.21 (s, 2H, OC**H**$_2$Ph), 6.13 (s, 1H,

C=C**H**), 7.67 (d, *J* = 8.1 Hz, 2H, Ar), 7.75 (d, *J* = 8.2 Hz, 2H, Ar); ^{13}C NMR (DMSO–d6, 75.5 MHz, 25°C): δ = 73.2, 111.1, 124.6 (q, *J* = 271.9 Hz), 125.6 (q, *J* = 3.8 Hz), 128.5, 128.8 (q, *J* = 31.6 Hz), 142.0, 154.1, 164.4, 165.7; ^{19}F NMR (DMSO–d6, 200 MHz, 25°C): δ = –61.0; HRMS–ESI: *m/z* $[M]^-$ calcd for $C_{12}H_8F_3O_5$: 289.03293; found: 289.03313.

2-(2-methyl)benzyloxyfumaric acid (2h)

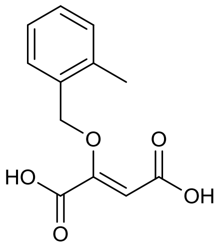

White solid. 700 mg (99% yield). Mp 132–133°C (diethyl ether); ^{1}H NMR (DMSO–d6, 300 MHz, 25°C): δ = 2.28 (s, 3H, Ar–C**H**$_3$), 5.11 (s, 2H, OC**H**$_2$Ph), 6.04 (s, 1H, C=C**H**), 7.15–7.29 (m, 3H, Ar), 7.40 (d, *J* = 7.4 Hz, 1H, Ar); ^{13}C NMR (DMSO–d6, 75.5 MHz, 25°C): δ = 18.3, 72.0, 109.6, 125.7, 128.3, 129.1, 130.0, 134.5, 136.9, 153.6, 164.1, 165.3; HRMS–ESI: *m/z* $[M]^-$ calcd for $C_{12}H_{11}O_5$: 235.0612; found: 235.06138.

2-(3-methyl)benzyloxyfumaric acid (2i)

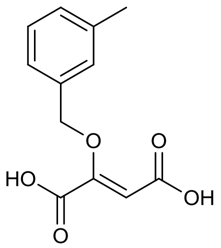

White solid. 665 mg (94% yield). Mp 132–133°C (diethyl ether); ^{1}H NMR (DMSO–d6, 300 MHz, 25°C): δ = 2.30 (s, 3H, Ar–C**H**$_3$), 5.06 (s, 2H, OC**H**$_2$Ph), 6.07 (s, 1H, C=C**H**), 7.12–7.29 (m, 4H, Ar); ^{13}C NMR (DMSO–d6, 75.5 MHz, 25°C): δ = 21.0, 73.7, 110.4, 125.0, 128.2, 128.5, 128.7, 136.5, 137.4, 153.6, 164.1, 165.3; HRMS–ESI: *m/z* $[M]^-$ calcd for $C_{12}H_{11}O_5$: 235.0612; found: 235.06149.

2-(4-methyl)benzyloxyfumaric acid (2j)

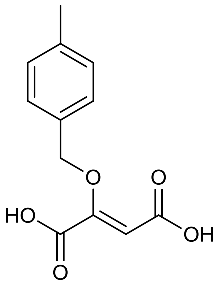

White solid. 641 mg (91% yield). Mp 138–139°C (diethyl ether); ^{1}H NMR (DMSO–d6, 300 MHz, 25°C): δ = 2.30 (s, 3H, Ar–C**H**$_3$), 5.06 (s, 2H, OC**H**$_2$Ph), 6.04 (s, 1H, C=C**H**), 7.17 (d, *J* = 7.9 Hz, 2H, Ar), 7.29 (d, *J* = 7.9 Hz, 2H, Ar); ^{13}C NMR (DMSO–d6, 75.5 MHz, 25°C): δ = 20.9, 73.6, 110.3, 128.2, 129.0, 133.7, 137.5, 153.7, 164.2, 165.4; HRMS–ESI: *m/z* $[M]^-$ calcd for $C_{12}H_{11}O_5$: 235.0612; found: 235.06133.

Enzyme expression, purification and kinetic characterization

The MAL mutant enzymes, L384A and L384G, were overproduced in *E. coli* TOP10 cells using the pBADN expression system.[2] Briefly, fresh TOP10 cells containing the appropriate expression plasmid were collected from a LB/Ap plate to inoculate LB/Ap medium (5 mL) and grown overnight at 37°C. A sufficient quantity of the culture was used to inoculate 500 mL fresh auto-induction (ZYM) medium (10 g/L trypton, 5 g/L yeast extract, 25 mM Na_2HPO_4, 25 mM KH_2PO_4, 5 mM Na_2SO_4, pH 6.7), containing 0.5% (v/v) glycerol, 0.05% (w/v) glucose, 2 mM $MgSO_4$, 100 µg/mL ampicillin (Ap), and 0.05% (w/v) arabinose.[2] Cultures were grown for 40 h at room temperature with vigorous shaking (170 rpm). The cells were harvested by centrifugation (6000 × g, 45 min) and cell pellets were stored at -20°C until needed.

Protein purification was performed using an immobilized metal affinity chromatography procedure as previously described,[3] with the minor modification of Tris-HCl buffer (50 mM, pH 8) being used in combination with all other buffer components instead of phosphate buffer. The buffer (Tris-HCl) was removed from each enzyme solution to avoid product contamination. This

was achieved by using PD-10 desalting columns to recover the protein in 2 mM $MgCl_2$ and 0.1 mM KCl at pH 8. Proteins were stored at 4°C until further needed and the protein concentrations were determined by the method of Waddell.[4]

The L384A or L384G mutant catalyzed amination of **2a** was monitored by following the depletion of substrate at 285 nm at room temperature. To determine kinetic parameters for the enzyme-catalyzed amination of **2a**, the **2a** concentration was varied from 0-100 mM in 20 mM $MgCl_2$ with a constant NH_4Cl concentration of 2.5 M at pH 9. Alternatively, the concentration of NH_4Cl was varied from 0-2.5 M in 20 mM $MgCl_2$ with a constant **2a** concentration of 50 mM for L384G and 100 mM for L384A at pH 9. The reactions were performed with 150 µg enzyme (0.5 µg/µL) in a final volume of 300 µL. All substrate stock solutions were prepared in 20 mM $MgCl_2$, and the pH was adjusted to 9.0. The molar absorption coefficient of **2a** was determined as 378 $M^{-1}cm^{-1}$ in the same solution. Initial rates were determined in triplicate and kinetic parameters were obtained by fitting the data to the Michaelis-Menten equation using Sigmaplot 12.1 (Systat Software).

Progress curves for the L384A- and L384G-catalyzed amination of substrate 2a

Reaction mixtures (6 mL) consisted of 5 M NH_4Cl and 50 mM **2a** in 20 mM $MgCl_2$ (pH 9). The reactions were started by the addition of freshly purified enzyme (0.01 mol%), and reaction mixtures were incubated at room temperature. Aliquots (1 mL) of the reaction mixtures were withdrawn at 15 min, 45 min, 90 min, 180 min, 6 h and 24 h after the addition of enzyme. For each sample, the reaction was stopped by incubating the reaction mixture at 100°C for 5 min. Each sample was dried *in vacuo*, followed by suspending the resulting residue in ~10 mL of

water for lyophilization. This process was repeated 5 times in order to remove the excess of ammonia to enable analysis by ^{1}H NMR spectroscopy. The final residue was dissolved in 800 µL D_2O and 10 µL 10 M NaOH. The conversion of substrate to product was estimated by integration of the respective substrate and product signals.

Procedure for monitoring the amination of various 2-benzyloxyfumarate derivatives

Reaction mixtures (1 mL) consisted of 5 M NH_4Cl and 50 mM **2a-2j** in 20 mM $MgCl_2$ (pH 9). The reactions were started by the addition of freshly purified enzyme (0.01 or 0.05 mol%), and reaction mixtures were incubated at room temperature for either 3 h or 24 h. For each sample, the reaction was stopped by incubating the reaction mixture at 100°C for 5 min. Each sample was dried *in vacuo*, followed by suspending the resulting residue in ~10 mL of water for lyophilization. This process was repeated 5 times in order to remove the excess of ammonia to enable analysis by ^{1}H NMR spectroscopy. For ^{1}H NMR spectroscopic analysis, the final residue was dissolved in 800 µL D_2O and 10 µL 10 M NaOH. The conversion of substrate to product was estimated by integration of the respective substrate and product signals.

Enzymatic synthesis, purification and characterization of amino acid products 1a-1f, 1h, and 1i

Reaction mixtures (15 mL) consisted of 5 M NH_4Cl and 50 mM fumarate derivative (**2a-2f**, **2h**, or **2i**; 167-218 mg, 0.75 mmol) in 20 mM $MgCl_2$ (pH 9). The reactions were started by the addition of freshly purified MAL(L384G) enzyme (0.01 or 0.05 mol%), and reaction mixtures were incubated at room temperature for 24 h. Each reaction mixture was lyophilized, suspended

in 15 mL water and acidified with conc. HCl to pH <1. The reaction mixture was loaded onto a column packed with cation-exchange resin (50 g of Dowex 50W X8, 50-100 mesh), which was pre-treated with 2 M aqueous ammonia (4 column volumes), 1 M HCl (2 column volumes) and distilled water (4 column volumes). The column was washed with water (1 column volume) and the product was eluted with 2 M aqueous ammonia (2 column volumes). The ninhydrin-positive fractions were collected and lyophilized (3 times) to yield the products as the bisammonium salt (white powder).

L-*threo*-3-benzyloxyaspartate (L-TBOA) (1a)

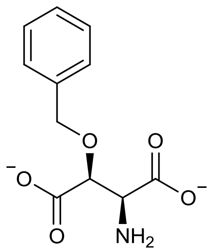

White powder. 139 mg (78% yield). ^{1}H NMR (D_2O, 500 MHz, 25°C): δ = 4.02 (d, J = 2.3 Hz, 1H, C**H**NH$_2$), 4.35 (d, J = 2.3 Hz, 1H, C**H**OCH$_2$Ph), 4.49 (d, J = 11.6 Hz, 1H, OC**H**$_2$Ph), 4.74 (d, J = 11.6 Hz, 1H, OC**H**$_2$Ph), 7.38–7.45 (m, 5H, Ar); ^{13}C NMR (D_2O, 75.5 MHz, 25°C): δ = 56.4, 72.4, 77.1, 128.2, 128.4, 128.5, 136.8, 198.7, 200.7; HRMS–ESI: *m/z* [M]$^-$ calcd for $C_{11}H_{12}O_5N_1$: 238.0721; found: 238.0722.

***threo*-3-(2-fluoro)benzyloxyaspartate (1b)**

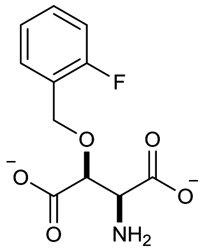

White powder. 115 mg (60% yield). ^{1}H NMR (D_2O, 500 MHz, 25°C): δ = 3.98 (d, J = 1.4 Hz, 1H, C**H**NH$_2$), 4.31 (d, J = 1.6 Hz, 1H, C**H**OCH$_2$Ph), 4.56 (d, J = 11.9 Hz, 1H, OC**H**$_2$Ph), 4.76 (d, J = 11.9 Hz, 1H, OC**H**$_2$Ph), 7.09–7.15 (m, 1H, Ar), 7.17–7.22 (m, 1H, Ar), 7.36–7.41 (m, 2H, Ar); ^{13}C NMR (D_2O, 75.5 MHz, 25°C): δ = 56.4, 66.1 (d, J = 3.6 Hz), 77.1, 115.3 (d, J = 21.3 Hz), 123.4 (d, J = 14.9 Hz), 124.2

(d, J = 3.6 Hz), 130.5 (d, J = 8.3 Hz), 131.2 (d, J = 4.1 Hz), 160.9 (d, J = 246.0 Hz), 171.1, 175.5; ^{19}F NMR (D_2O, 200 MHz, 25°C): δ = –118.9; HRMS–ESI: *m/z* $[M]^-$ calcd for $C_{11}H_{11}O_5N_1F_1$: 256.06267; found: 256.06287.

threo-3-(3-fluoro)benzyloxyaspartate (1c)

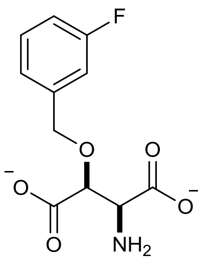

White powder. 118 mg (61% yield). ^{1}H NMR (D_2O, 500 MHz, 25°C): δ = 3.97 (d, J = 2.1 Hz, 1H, C**H**NH_2), 4.31 (d, J = 2.1 Hz, 1H, C**H**OCH_2Ph), 4.48 (d, J = 12.1 Hz, 1H, OC**H**$_2$Ph), 4.74 (d, J = 12.0 Hz, 1H, OC**H**$_2$Ph), 7.06–7.23 (m, 3H, Ar), 7.38–7.43 (m, 1H, Ar); ^{13}C NMR ($CDCl_3$, 75.5 MHz, 25°C): δ = 56.6, 71.5 (d, J = 1.9 Hz), 77.8, 114.8 (d, J = 21.4 Hz, two overlapping signals responsible for two carbon atoms), 123.9 (d, J = 2.8 Hz), 130.1 (d, J = 8.4 Hz), 139.5 (d, J = 7.4 Hz), 162.5 (d, J = 243.0 Hz), 172.0, 175.8; ^{19}F NMR (D_2O, 200 MHz, 25°C): δ = –114.0; HRMS–ESI: *m/z* $[M]^-$ calcd for $C_{11}H_{11}O_5N_1F$: 256.06267; found: 256.06288.

threo-3-(4-fluoro)benzyloxyaspartate (1d)

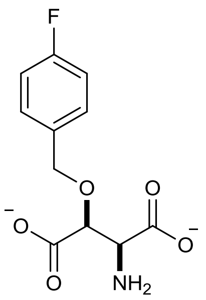

White powder. 110 mg (57% yield). ^{1}H NMR (D_2O, 500 MHz, 25°C): δ = 3.97 (d, J = 2.1 Hz, 1H, C**H**NH_2), 4.28 (d, J = 2.0 Hz, 1H, C**H**OCH_2Ph), 4.43 (d, J = 11.5 Hz, 1H, OC**H**$_2$Ph), 4.67 (d, J = 11.6 Hz, 1H, OC**H**$_2$Ph), 7.07–7.14 (m, 2H, Ar), 7.32–7.39 (m, 2H, Ar); ^{13}C NMR (D_2O, 75.5 MHz, 25°C): δ = 56.4, 71.6, 77.1, 115.2 (d, J = 21.5 Hz), 130.3 (d, J = 8.5 Hz), 132.6 (d, J = 3.1 Hz), 162.3 (d, J = 244.0 Hz), 171.3, 171.6; ^{19}F NMR (D_2O, 200 MHz, 25°C): δ = –114.8; HRMS–ESI: *m/z* $[M]^-$ calcd for $C_{11}H_{11}O_5NF$: 256.06267; found: 256.06288.

threo-3-(2-trifluoromethyl)benzyloxyaspartate (1e)

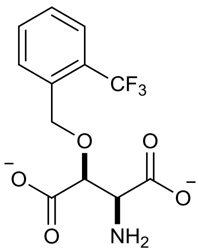

White powder. 134 mg (58% yield). ^{1}H NMR (D_2O, 500 MHz, 25°C): δ = 3.56 (d, J = 2.4 Hz, 1H, C**H**NH_2), 4.17 (d, J = 2.4 Hz, 1H, C**H**OCH_2Ph), 4.62 (d, J = 13.4 Hz, 1H, OC**H**$_2$Ph), 4.88 (d, J = 13.5 Hz, 1H, OC**H**$_2$Ph), 7.48 (t, J = 7.5 Hz, 1H, Ar), 7.62–7.75 (m, 3H, Ar); ^{13}C NMR (D_2O, 75.5 MHz, 25°C): δ = 57.6, 67.8, 79.9, 123.8 (q, J = 272.4 Hz), 125.6 (q, J = 5.7 Hz), 127.2 (q, J = 30.8 Hz), 127.9, 130.0, 132.3, 135.5 (q, J = 3.1 Hz), 175.7, 176.8; ^{19}F NMR (D_2O, 200 MHz, 25°C): δ = –59.3; HRMS–ESI: *m/z* $[M]^-$ calcd for $C_{12}H_{11}O_5NF_3$: 306.05948; found: 306.05978.

threo-3-(3-trifluoromethyl)benzyloxyaspartate (1f)

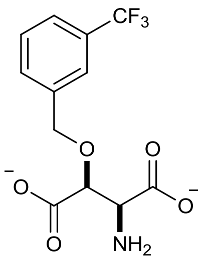

White powder. 177 mg (77% yield). ^{1}H NMR (D_2O, 500 MHz, 25°C): δ = 3.56 (d, J = 2.4 Hz, 1H, C**H**NH_2), 4.18 (d, J = 2.4 Hz, 1H, C**H**OCH_2Ph), 4.43 (d, J = 12.2 Hz, 1H, OC**H**$_2$Ph), 4.73 (d, J = 12.2 Hz, 1H, OC**H**$_2$Ph), 7.54 (t, J = 7.7 Hz, 1H, Ar), 7.60 (d, J = 7.6 Hz, 1H, Ar), 7.64 (d, J = 7.7 Hz, 1H, Ar), 7.69 (s, 1H, Ar); ^{13}C NMR (D_2O, 75.5 MHz, 25°C): δ = 58.3, 71.4, 81.7, 124.1 (q, J = 271.0 Hz), 124.5 (q, J = 3.9 Hz), 124.7 (q, J = 3.9 Hz) 129.3 (q, J = 32.0 Hz), 131.6, 131.7, 138.6, 177.9, 179.0; ^{19}F NMR (D_2O, 200 MHz, 25°C): δ = –62.3; HRMS–ESI: *m/z* $[M]^-$ calcd for $C_{12}H_{11}O_5NF_3$: 306.05948; found: 306.05966.

***threo*-3-(2-methyl)benzyloxyaspartate (1h)**

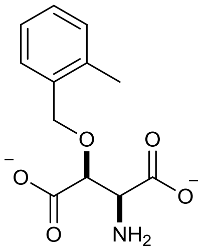

White powder. 135 mg (71% yield). ^{1}H NMR (D_2O, 500 MHz, 25°C): δ = 2.30 (s, 3H, Ar–C**H**$_3$), 3.54 (d, *J* = 2.3 Hz, 1H, C**H**NH$_2$), 4.16 (d, *J* = 2.3 Hz, 1H, C**H**OCH$_2$Ph), 4.40 (d, *J* = 11.5 Hz, 1H, OC**H**$_2$Ph), 4.69 (d, *J* = 11.5 Hz, 1H, OC**H**$_2$Ph), 7.22–7.30 (m, 3H, Ar), 7.34 (d, *J* = 7.3 Hz, 1H, Ar); ^{13}C NMR (D_2O, 75.5 MHz, 25°C): δ = 20.3, 57.5, 72.2, 79.6, 125.3, 128.5, 128.6, 128.9, 137.3, 138.6, 175.5, 177.0; HRMS–ESI: *m/z* [M]$^-$ calcd for $C_{12}H_{14}O_5N$: 252.08775; found: 252.08801.

***threo*-3-(3-methyl)benzyloxyaspartate (1i)**

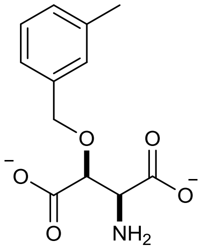

White powder. 139 mg (73% yield). ^{1}H NMR (D_2O, 500 MHz, 25°C): δ = 2.35 (s, 3H, Ar–C**H**$_3$), 3.62 (d, *J* = 2.3 Hz, 1H, C**H**NH$_2$), 4.21 (d, *J* = 2.3 Hz, 1H, C**H**OCH$_2$Ph), 4.37 (d, *J* = 11.7 Hz, 1H, OC**H**$_2$Ph), 4.64 (d, *J* = 11.7 Hz, 1H, OC**H**$_2$Ph), 7.20 (d, *J* = 6.9 Hz, 1H, Ar), 7.21 (d, *J* = 6.5 Hz, 1H, Ar), 7.30 (s, 1H, Ar), 7.31 (t, *J* = 7.6 Hz, 1H, Ar); ^{13}C NMR (D_2O, 75.5 MHz, 25°C): δ = 20.3, 56.5, 72.3, 77.4, 125.3, 128.5, 128.8, 129.0, 136.9, 138.7, 171.5, 175.9; HRMS–ESI: *m/z* [M]$^-$ calcd for $C_{12}H_{14}O_5N_1$: 252.08775; found: 252.08785.

Diastereomeric and enantiomeric excess determination of enzymatically obtained TBOA (1a) and derivatives 1b-f,h,i.

Synthesis of racemic 1

Racemic (±)-*threo*-benzyloxyasparate (**1a**, TBOA), (±)-*erythro*-benzyloxyasparate (**1a**, EBOA), and derivatives **1c**,**f**,**i** were synthesized to serve as reference compounds for diasteromeric excess (*de*) determination (^{1}H NMR) and enantiomeric excess (*ee*) determination (chiral phase HPLC) of enzymatically obtained enantioenriched TBOA (**1a**) and derivatives **1c**,**f**,**i** (*de* of **1b-f**,**h** and *ee* of **1b**,**d**,**e**,**h** were tentatively assigned). Furthermore, the absolute configuration of enzymatically obtained (MAL) TBOA (**1a**) was determined (HPLC and optical rotation) while the absolute configurations of all other derivatives **1b-f**,**h**,**i** were tentatively assigned.

Dilithium salts of racemic TBOA ((±)-*threo*-**1a**) and EBOA ((±)-*erythro*-**1a**) and derivatives **1c**,**f**,**i** were synthesized according to the synthesis route as depicted in Scheme S1. Racemic *threo*-dimethyl 2-(benzyloxycarbonylamino)-3-hydroxysuccinate ((±)-*threo* **5**) was readily obtained at gram scale by a literature precedented aminohydroxylation of dimethyl fumarate.[5] A sodium hydride initiated Williamson ether synthesis of (±)-*threo*-**5** with benzyl bromide **6a**, and *meta*-substituted derivatives hereof (**6c**,**f**,**i**), in DMF at −20°C yielded products **7a**,**c**,**f**,**i**, respectively. Adducts **7a**,**c**,**f**,**i** were obtained as mixtures of (±)-*threo*- and (±)-*erythro*-isomers with ratios of ~90/10 as determined by ^{1}H NMR spectroscopy. Pure (±)-*threo*-isomers of **7a**,**c**,**i** (R = H, F, Me) were obtained by column chromatography on silica gel (eluted as first fraction) whereas isomers of the *m*-CF_3-derivative (**7f**, R = CF_3) could not be separated in this way. (±)-*Erythro* isomers of **7a**,**i** (R = H, Me), which eluted as second fractions, were collected but could

not be isolated in pure form as they co-eluted with the remainder of the (±)-*threo*-isomers of **7a**,**i**. At best, a mixture of (±)-*threo*/*erythro*-**7a**,**i** (R = H, Me) 35/65 was obtained.

Pure (±)-*threo*-isomers of **7a**,**c**,**i** (R = H, F, Me) and the mixtures of (±)-*threo*/*erythro*-isomers of **7a**,**i** (R = H, Me) were submitted for the next synthesis step in separate experiments (in case of *m*-CF_3-derivative **7f**, the *threo*/*erythro* 90/10 mixture was used). Deprotection of the amine functionality of **7a**,**c**,**f**,**i** by hydrogenation with Pd/C (10%) and H_2 in MeOH afforded amines **8a**,**c**,**f**,**i**. Finally, hydrolysis of **8a**,**c**,**f**,**i** in the presence of ~6 equivalents of LiOH in D_2O gave desired racemic (±)-*threo*-**1a** (R = H, TBOA) and derivatives **1c**,**i** (*threo*/*erythro* ratios of 99/1), racemic (±)-*erythro*-**1a** (R = H, EBOA) and (±)-*erythro*-**1i** (R = Me) as mixtures with their respective (±)-*threo*-isomers (ratios of ~35/65 *threo*/*erythro*), and (±)-*threo*-**1f** (R = CF_3) as a mixture with its (±)-*erythro*-isomer (ratio ~90/10). Products **1a**,**c**,**f**,**i** were obtained as dilithium salts.

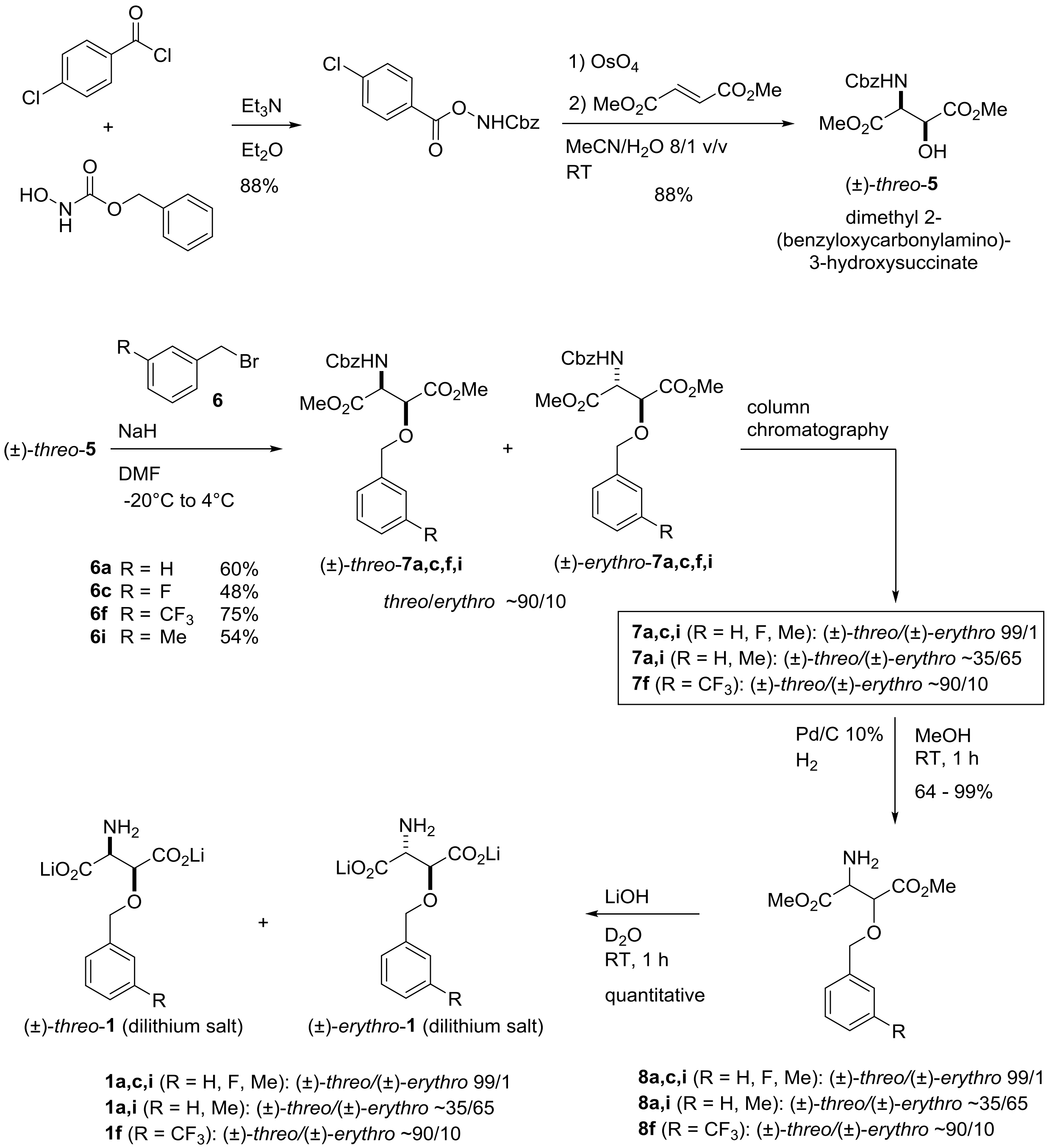

Scheme S1. Chemical synthesis of dilithium salts of racemic benzyloxy aspartate **1a** (R = H) and derivatives **1c**,**f**,**i** (R = F, CF_3, Me).

General procedure for the synthesis of 7a,c,f,i. A mixture of (±)-*threo*-**5**[5] (1 eq) and benzyl bromide **6** (4 eq), or a derivative hereof, in DMF was stirred and cooled to −20°C. NaH (1 eq, 60% dispersion in mineral oil) was added and the mixture was stirred at −20°C. Reaction progress was monitored by thin layer chromatography (silica gel, EtOAc/petroleum ether 1/1, visualization: $KMnO_4$). After 4 h, the mixture was allowed to slowly warm up to 4°C after which it was stirred overnight at this temperature. Water was added and stirring maintained for 10 min. The mixture was extracted with EtOAc (3 times). The combined organic layers were washed with brine (3 times). The portions of brine were kept relatively small since product **7** dissolves in water to some extent. The organic layer was dried on $MgSO_4$ and concentrated *in vacuo* to yield a colourless viscous oil which was purified by column chromatography.

(±)-*Threo*- and *erythro*-dimethyl 2-(benzyloxy)-3-(((benzyloxy)carbonyl)amino)succinate (*threo*- and *erythro*-7a)

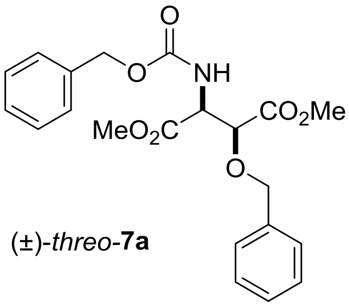

(±)-*threo*-**7a**

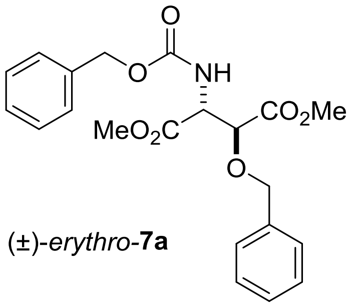

(±)-*erythro*-**7a**

(±)-*Threo*-**5** (400 mg, 1.28 mmol), benzyl bromide **6a** (879 mg, 5.14 mmol), and NaH (30.7 mg, 1.28 mmol) in DMF (15 mL) gave pure (±)-*threo*-**7a** (218 mg, 0.54 mmol, 42%) and a mixture of *threo*-**7a** and *erythro*-**7a** with a ~35/65 ratio (73 mg, 18%) after column chromatography

(silica gel, EtOAc/petroleum ether 1/5 → 1/4). Portions used during work up procedure: water (30 mL), EtOAc (3×30 mL), brine (3×15 mL).

(±)-*Threo*-7a: ^{1}H NMR (500 MHz, $CDCl_3$, 25°C) δ 7.40 – 7.31 (m, 9H), 7.28 (d, *J* = 2.1 Hz, 1H), 5.61 (d, *J* = 9.8 Hz, 1H), 5.13 (q, AB system, J_{AB} = 12.5 Hz, $\nu_A - \nu_B$ = 5.8 Hz, 2H), 4.89 (dd, *J* = 9.8, 1.9 Hz, 1H), 4.84 (d, *J* = 11.8 Hz, 1H), 4.54 (d, *J* = 1.9 Hz, 1H), 4.41 (d, *J* = 11.8 Hz, 1H), 3.76 (s, 3H), 3.65 (s, 3H); ^{13}C NMR (125 MHz, $CDCl_3$, 25°C) δ 169.49, 169.33, 156.06, 136.42, 136.09, 128.40, 128.38, 128.38, 128.21, 128.21, 128.17, 128.17, 128.03, 127.85, 127.85, 76.53, 72.78, 67.07, 56.36, 52.61, 52.39; HRMS (ESI): *m*/*z* = 402.15480 $[M+H]^+$ (calcd. 402.15473 for $C_{21}H_{24}NO_7$)

(±)-*Erythro*-7a: ^{1}H NMR (500 MHz, $CDCl_3$, 25°C) δ 7.41 – 7.27 (m, 10H), 5.60 (d, *J* = 8.5 Hz, 1H), 5.07 (q, AB system, J_{AB} = 12.2 Hz, $\nu_A - \nu_B$ = 15.1 Hz, 2H), 4.95 (dd, *J* = 8.5, 3.0 Hz, 1H), 4.88 (d, *J* = 11.9 Hz, 1H), 4.51 (d, *J* = 11.9 Hz, 1H), 4.34 (d, *J* = 3.0 Hz, 1H), 3.82 (s, 3H), 3.76 (s, 3H); ^{13}C NMR (125 MHz, $CDCl_3$, 25°C) δ 169.55, 168.81, 155.56, 136.59, 136.05, 128.49, 128.49, 128.47, 128.47, 128.24, 128.18, 128.10, 128.10, 128.10, 128.10, 77.39, 73.06, 67.11, 55.92, 52.78, 52.29; HRMS (ESI): *m*/*z* = 402.15470 $[M+H]^+$ (calcd. 402.15473 for $C_{21}H_{24}NO_7$)

(±)-*Threo*-dimethyl 2-(3-fluorobenzyloxy)-3-(((benzyloxy)carbonyl)amino)-succinate (*threo*-7c)

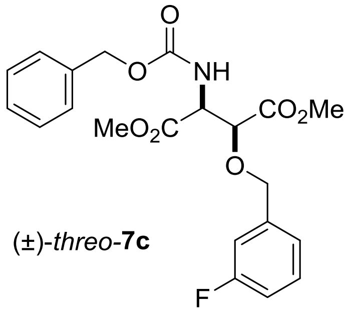

(±)-*threo*-**7c**

(±)-*Threo*-**5** (250 mg, 0.80 mmol), 3-fluorobenzyl bromide **6c** (607 mg, 3.21 mmol), and NaH (19.2 mmol, 0.80 mmol) in DMF (10 mL) gave pure (±)-*threo*-**7c** (162 mg, 0.39 mmol, 48%) after column chromatography (silica gel, EtOAc/petroleum ether 1/4). Portions used during work up procedure: water (20 mL), EtOAc (3×25 mL), brine (3×15 mL). ^{1}H NMR (500 MHz, $CDCl_3$, 25°C) δ 7.38 – 7.27 (m, 6H), 7.02 – 6.96 (m, 3H), 5.56 (d, *J* = 9.5 Hz, 1H), 5.11 (s, 2H), 4.90 (d, *J* = 9.5 Hz, 1H), 4.81 (d, *J* = 12.1 Hz, 1H), 4.51 (s, 1H), 4.39 (d, *J* = 12.1 Hz, 1H), 3.75 (s, 3H), 3.70 (s, 3H); ^{13}C NMR (125 MHz, $CDCl_3$, 25°C) δ 169.21, 169.17, 162.74 (d, *J* = 246.1 Hz), 155.96, 139.15 (d, *J* = 7.3 Hz), 136.08, 129.83 (d, *J* = 8.2 Hz), 128.31, 128.31, 127.95, 127.77, 127.77, 123.31 (d, *J* = 2.8 Hz), 114.88 (d, *J* = 21.1 Hz), 114.61 (d, *J* = 21.8 Hz), 76.98, 71.93, 67.01, 56.31, 52.55, 52.27; HRMS (ESI): *m*/*z* = 420.14545 $[M+H]^+$ (calcd. 420.14531 for $C_{21}H_{23}O_7NF$)

(±)-*Threo*- and *erythro*-dimethyl 2-(3-trifluoromethylbenzyloxy)-3-(((benzyloxy)carbonyl)-amino)succinate (*threo*- and *erythro*-7f)

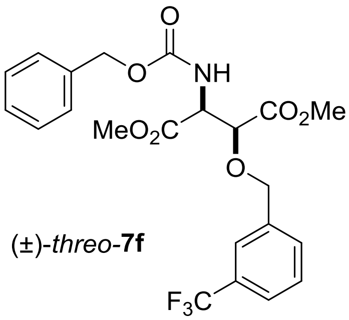

(±)-*threo*-**7f**

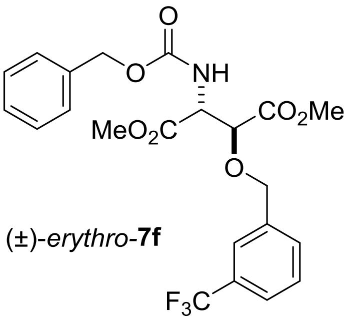

(±)-*erythro*-**7f**

(±)-*Threo*-**5** (250 mg, 0.80 mmol), 3-trifluoromethylbenzyl bromide **6f** (767 mg, 3.21 mmol), and NaH (19.2 mmol, 0.80 mmol) in DMF (10 mL) gave a mixture of *threo*-**7f** and *erythro*-**7f** (280 mg, 0.60 mmol, 75%, ratio 90/10) after column chromatography (silica gel, EtOAc/petroleum ether 2/9 → 1/4, the two diastereomers appeared to be inseparable under these chromatography conditions). Portions used during work up procedure: water (20 mL), EtOAc (3×25 mL), brine (3×15 mL). ^{1}H NMR (500 MHz, $CDCl_3$, 25°C) δ 7.59 – 7.55 (m, 1H), 7.52 (s, 1H), 7.47 – 7.44 (m, 2H), 7.37 – 7.29 (m, 5H), 5.55 (d, *J* = 9.8 Hz, 1H), 5.11 (s, 2H), 4.92 (dd, *J* = 9.8, 1.7 Hz, 1H), 4.87 (d, *J* = 12.0 Hz, 1H), 4.56 (d, *J* = 1.7 Hz, 1H), 4.45 (d, *J* = 12.0 Hz, 1H), 3.75 (s, 3H), 3.69 (s, 3H); ^{13}C NMR (125 MHz, $CDCl_3$, 25°C) δ 169.21, 169.12, 155.96, 137.67, 136.05, 131.10, 130.71 (q, *J* = 32.3 Hz), 128.82, 128.32, 128.32, 127.97, 127.78, 127.78, 124.79 (q, *J* = 3.7 Hz), 124.44 (q, *J* = 3.5 Hz), 123.91 (q, *J* = 272.3 Hz), 77.43, 72.08, 67.04, 56.32, 52.57, 52.30; HRMS (ESI): *m*/*z* = 470.14229 $[M+H]^+$ (calcd. 470.14211 for $C_{22}H_{23}O_7NF_3$)

(±)-*Threo*- and *erythro*-dimethyl 2-(3-methylbenzyloxy)-3-(((benzyloxy)carbonyl)amino)-succinate (*threo*- and *erythro*-7i).

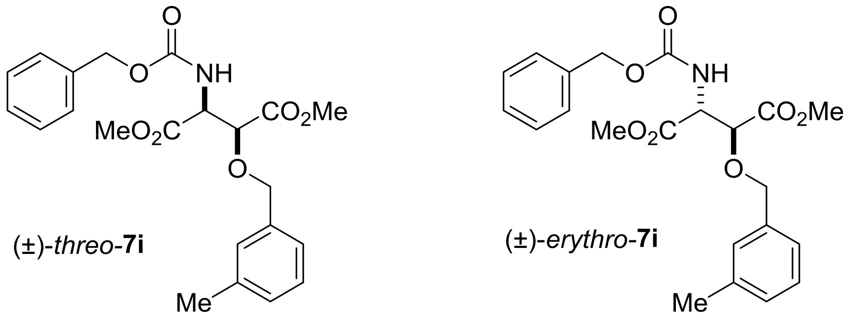

(±)-*Threo*-**5** (400 mg, 1.28 mmol), 3-methylbenzyl bromide **6i** (951 mg, 5.14 mmol), and NaH (30.7 mmol, 1.28 mmol) in DMF (10 mL) gave pure (±)-*threo*-**7i** (250 mg, 0.60 mmol, 47%) and a mixture of *threo*-**7i** and *erythro*-**7i** with a ~35/65 ratio (35 mg, 8.4×10^{-2} mmol, 7%) after column chromatography (silica gel, EtOAc/petroleum ether 1/5 → 1/4). Portions used during work up procedure: water (25 mL), EtOAc (3×25 mL), brine (3×15 mL).

(±)-*Threo*-7i: ^{1}H NMR (500 MHz, $CDCl_3$, 25°C) δ 7.38 – 7.28 (m, 5H), 7.22 (dd, *J* = 7.6, 7.6 Hz, 1H), 7.12 (d, *J* = 7.6 Hz, 1H), 7.06 (s, 1H), 7.04 (d, *J* = 7.6 Hz, 1H), 5.58 (d, *J* = 9.8 Hz, 1H), 5.13 (q, AB system, J_{AB} = 12.5 Hz, $\nu_A - \nu_B$ = 6.3 Hz, 2H), 4.86 (dd, *J* = 9.8, 2.0 Hz, 1H), 4.78 (d, *J* = 11.7 Hz, 1H), 4.51 (d, *J* = 2.0 Hz, 1H), 4.35 (d, *J* = 11.7 Hz, 1H), 3.75 (s, 3H), 3.63 (s, 3H), 2.34 (s, 3H); ^{13}C NMR (125 MHz, $CDCl_3$, 25°C) δ 169.45, 169.25, 155.99, 137.93, 136.26, 136.06, 128.90, 128.83, 128.32, 128.32, 128.20, 127.94, 127.76, 127.76, 125.24, 76.44, 72.76, 66.97, 56.31, 52.50, 52.29, 21.20; HRMS (ESI): *m*/*z* = 416.17047 $[M+H]^+$ (calcd. 416.17038 for $C_{22}H_{26}O_7N$)

(±)-*Erythro*-7i: ^{1}H NMR (500 MHz, $CDCl_3$, 25°C) δ 7.39 – 7.28 (m, 5H), 7.20 (dd, J = 7.4, 7.4 Hz, 1H), 7.11 (s, 1H), 7.10 (d, J = 7.4 Hz, 1H), 7.09 (d, J = 7.4 Hz, 1H), 5.57 (d, J = 8.8 Hz, 1H), 5.07 (q, AB system, J_{AB} = 12.2 Hz, $\nu_A - \nu_B$ = 22.5 Hz, 2H), 4.93 (dd, J = 8.8, 3.0 Hz, 1H), 4.83 (d, J = 11.9 Hz, 1H), 4.45 (d, J = 3.0 Hz, 1H), 4.30 (d, J = 11.9 Hz, 1H), 3.80 (s, 3H), 3.74 (s, 3H), 2.31 (s, 3H); ^{13}C NMR (125 MHz, $CDCl_3$, 25°C) δ 169.46, 168.74, 155.47, 138.04, 136.35, 135.94, 128.77, 128.77, 128.36, 128.36, 128.27, 128.05, 127.92, 127.76, 125.13, 77.18, 72.95, 66.98, 55.75, 52.63, 52.15, 21.17 HRMS (ESI): m/z = 416.17054 $[M+H]^+$ (calcd. 416.17038 for $C_{22}H_{26}O_7N$)

General procedure for the synthesis of 8a,c,f,i. In separate experiments, a solution of (±)-*threo*-**7a**,**c**,**f**,**i**, or a mixture of (±)-*threo*- and *erythro*-isomers of **7a** or **7i**, in methanol was transferred into a flask that was charged with Pd/C 10%. The atmosphere in the flask was replaced by a hydrogen atmosphere (balloon, ~1 atm) and the mixture was stirred at room temperature until complete conversion of starting material was indicated by thin layer chromatography (silica gel, EtOAc/petroleum ether 1/1, R_f of starting material **7**: ~0.65; R_f of product **8**: ~0.20). The solution was filtered over celite and the filtrate concentrated *in vacuo* to yield products **8a**,**c**,**f**,**i** as colourless oils.

(±)-*Threo*-dimethyl 3-(benzyloxy)aspartate (*threo*-8a)

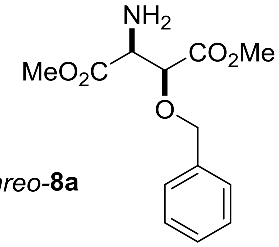

(±)-*threo*-**8a**

(±)-*Threo*-**7a** (100 mg, 2.5×10^{-1} mmol) and Pd/C 10% (5.0 mg, 4.7×10^{-3} mmol) in MeOH (4 mL) gave (±)-*threo*-**8a** (43 mg, 1.6×10^{-1} mmol, 64%) after purification by column chromatography (silica gel, EtOAc/petroleum ether 1/2 → 1/1). ^{1}H NMR (500 MHz, $CDCl_3$, 25°C) δ 7.35 – 7.24 (m, 5H), 4.84 (d, *J* = 11.9 Hz, 1H), 4.44 (d, *J* = 2.4 Hz, 1H), 4.41 (d, *J* = 11.9 Hz, 1H), 3.92 (b, 1 H), 3.82 (s, 3H), 3.62 (s, 3H), 2.19 (b, 2H); ^{13}C NMR (125 MHz, $CDCl_3$, 25°C) δ 172.09, 170.36, 136.74, 128.28, 128.28, 128.16, 128.16, 127.97, 77.83, 72.87, 56.80, 52.34, 52.32; HRMS (ESI): *m*/*z* = 268.11765 $[M+H]^+$ (calcd. 268.11795 for $C_{13}H_{18}O_5N$)

(±)-*Erythro*-dimethyl 3-(benzyloxy)aspartate (*erythro*-8a)

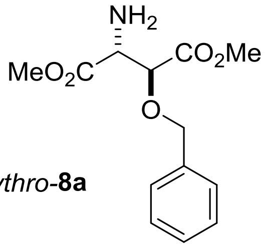

(±)-*erythro*-**8a**

A mixture of (±)-*threo*- and (±)-*erythro*-**7a** with ratio 35/65 (29 mg, 7.2×10^{-2} mmol) and Pd/C 10% (2.6 mg, 2.4×10^{-3} mmol) in MeOH (2 mL) gave a mixture of (±)-*threo*- and (±)-*erythro*-**8a**

with ratio 35/65 (17 mg, 6.4×10^{-2} mmol, 88%). Despite the presence of some impurities, no further purification was undertaken. (±)-*Erythro*: ^{1}H NMR (500 MHz, $CDCl_3$, 25°C) δ 7.38 – 7.24 (m, 5H), 4.84 (d, *J* = 11.5 Hz, 1H), 4.54 (d, *J* = 11.5 Hz, 1H), 4.40 (s, 1H), 4.10 (b, 1 H), 3.77 (s, 3H), 3.73 (s, 3H), 2.88 (b, 2H).

(±)-*Threo*-dimethyl 3-(3-fluorobenzyloxy)aspartate (*threo*-8c)

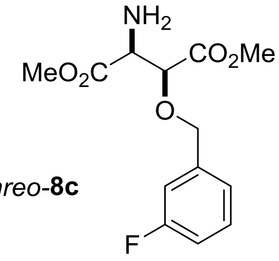

(±)-*threo*-**8c**

(±)-*Threo*-**7c** (100 mg, 2.4×10^{-1} mmol) and Pd/C 10% (3.2 mg, 3.0×10^{-3} mmol) in MeOH (3 mL) gave (±)-*threo*-**8c** (69 mg, 2.4×10^{-1} mmol, 99%). ^{1}H NMR (500 MHz, $CDCl_3$, 25°C) δ 7.30 – 7.26 (m, 1H), 7.04 – 6.95 (m, 3H), 4.83 (d, *J* = 12.1 Hz, 1H), 4.44 (d, *J* = 2.5 Hz, 1H), 4.43 (d, *J* = 12.1 Hz, 1H), 3.96 (d, *J* = 2.5 Hz, 1H), 3.82 (s, 3H), 3.68 (s, 3H), 2.63 (b, 2H); ^{13}C NMR (125 MHz, $CDCl_3$, 25°C) δ 172.33, 170.24, 162.83 (d, *J* = 246.1 Hz), 139.63 (d, *J* = 7.2 Hz), 129.70 (d, *J* = 8.1 Hz), 123.35 (d, *J* = 2.8 Hz), 114.78 (d, *J* = 21.2 Hz), 114.68 (d, *J* = 21.7 Hz), 78.52, 72.17 (d, *J* = 1.4 Hz), 57.01, 52.34, 52.27; HRMS (ESI): *m*/*z* = 286.10806 $[M+H]^+$ (calcd. 286.10853 for $C_{13}H_{17}O_5NF$)

(±)-*Threo*-dimethyl 3-(3-trifluoromethylbenzyloxy)aspartate (*threo*-8f)

(±)-*threo*-**8f**

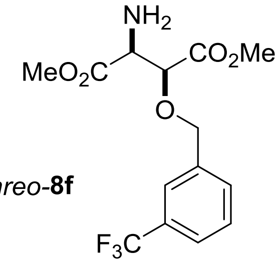

A mixture of (±)-*threo*- and (±)-*erythro*-**7f** with ratio 90/10 (100 mg, 2.1×10^{-1} mmol) and Pd/C 10% (3.2 mg, 3.0×10^{-3} mmol) in MeOH (4 mL) gave a mixture of (±)-*threo*- and (±)-*erythro*-**8f** with ratio 90/10 (70 mg, 2.1×10^{-1} mmol, 99%). (±)-*Threo*: ^{1}H NMR (500 MHz, $CDCl_3$, 25°C) δ 7.57 – 7.51 (m, 2H), 7.48 – 7.43 (m, 2H), 4.88 (d, *J* = 11.9 Hz, 1H), 4.48 (d, *J* = 11.9 Hz, 1H), 4.47 (s, 1H), 3.96 (s, 1H), 3.81 (s, 3H), 3.67 (s, 3H), 2.31 (b, 2H); ^{13}C NMR (125 MHz, $CDCl_3$, 25°C) δ 170.25, 170.25, 138.21, 131.12, 130.82 (q, *J* = 32.2 Hz), 128.80, 124.69 (q, *J* = 3.5 Hz), 124.49 (q, *J* = 3.5 Hz), 124.04 (q, *J* = 272.2 Hz), 79.23, 72.37, 57.15, 52.29, 52.23; HRMS (ESI): *m*/*z* = 336.10492 [M+H]$^{+}$ (calcd. 336.10533 for $C_{14}H_{17}O_5NF_3$)

(±)-*Threo*-dimethyl 3-(3-methylbenzyloxy)aspartate (*threo*-8i)

(±)-*threo*-**8i**

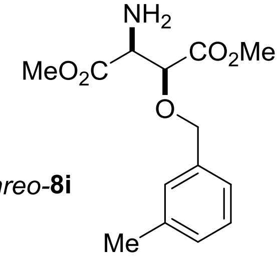

(±)-*Threo*-**7i** (136 mg, 3.3×10^{-1} mmol) and Pd/C 10% (5.0 mg, 4.7×10^{-3} mmol) in MeOH (4 mL) gave (±)-*threo*-**8i** (92 mg, 3.3×10^{-2} mmol, 99%). ^{1}H NMR (500 MHz, $CDCl_3$, 25°C) δ 7.22 (dd, *J* = 7.5, 7.5 Hz, 1H), 7.11 (d, *J* = 7.5 Hz, 1H), 7.09 (s, 1H), 7.07 (d, *J* = 7.5 Hz, 1H), 4.80 (d, *J* = 11.8 Hz, 1H), 4.43 (d, *J* = 2.9 Hz, 1H), 4.38 (d, *J* = 11.8 Hz, 1H), 3.92 (b, 1H), 3.82 (s, 3H), 3.63 (s, 3H), 2.35 (s, 3H), 2.01 (b, 2H); ^{13}C NMR (125 MHz, $CDCl_3$, 25°C) δ 172.84, 170.58, 137.79, 136.73, 128.81, 128.60, 128.10, 125.14, 78.28, 72.82, 57.13, 52.07, 52.07, 21.19; HRMS (ESI): *m*/*z* = 282.13329 $[M+H]^+$ (calcd. 282.13360 for $C_{14}H_{20}O_5N$)

(±)-*Erythro*-dimethyl 3-(3-methylbenzyloxy)aspartate (*erythro*-8i)

(±)-*erythro*-**8i**

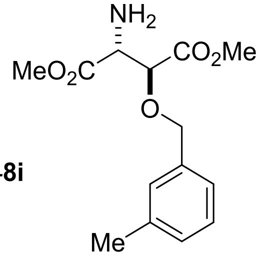

A mixture of (±)-*threo*- and (±)-*erythro*-**7i** with ratio 35/65 (31 mg, 7.4×10^{-2} mmol) and Pd/C 10% (1.6 mg, 1.5×10^{-3} mmol) in MeOH (2 mL) gave a mixture of (±)-*threo*- and (±)-*erythro*-**8i** with ratio 35/65 (19 mg, 6.8×10^{-2} mmol, 92%). Despite the presence of some impurities, no further purification was undertaken. (±)-*Erythro*: ^{1}H NMR (500 MHz, $CDCl_3$, 25°C) δ 7.23 (dd, *J* = 7.4, 7.4 Hz, 1H), 7.15 (s, 1H), 7.12 (d, *J* = 7.4 Hz, 1H), 7.11 (d, *J* = 7.4 Hz, 1H), 4.80 (d, *J* = 11.4 Hz, 1H), 4.47 (d, *J* = 11.4 Hz, 1H), 4.34 (b, 1H), 4.04 (b, 1H), 3.77 (s, 3H), 3.73 (s, 3H), 2.60 (b, 2H), 2.34 (s, 3H).

General procedure for the synthesis of dilithium salts of 1a,c,f,i. In separate experiments, (±)-*threo*-**8a**,**c**,**f**,**i**, and mixtures of (±)-*threo*- and *erythro*-isomers of **8a** or **8i**, were emulsified in a solution of LiOH (6 equivalents as compared to **8**) in D_2O. This mixture was stirred at room temperature until a homogeneous solution was obtained (~1 h). ^{1}H and ^{13}C NMR spectra recorded of the reaction mixture confirmed quantitative conversion of **8** into **1**. No further purification was undertaken and mixtures were used for exact mass spectroscopy and HPLC analysis.

Dilithium (±)-*threo*-3-(benzyloxy)aspartate (*threo*-1a)

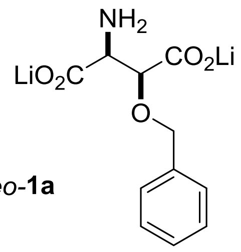

(±)-*threo*-**1a**

(±)-*Threo*-**8a** (35.0 mg, 1.3×10^{-1} mmol) in a solution of LiOH (18.6 mg, 7.8×10^{-1} mmol) in D_2O (0.9 mL) was converted quantitatively into (±)-*threo*-**1a**. ^{1}H NMR (500 MHz, D_2O, 25°C) δ 7.44 – 7.35 (m, 5H), 4.68 (d, *J* = 11.7 Hz, 1H), 4.40 (d, *J* = 11.7 Hz, 1H), 4.20 (d, *J* = 2.6 Hz, 1H), 3.56 (d, *J* = 2.6 Hz, 1H), signal of MeOH at δ 3.34; ^{13}C NMR (125 MHz, D_2O, 25°C) δ 180.09, 178.89, 138.30, 129.18, 129.06 (m), 129.18, 129.06 (m), 128.62 (m), 82.39 (m), 72.81 (m), 59.08 (m), signal of MeOH: δ 49.50; HRMS (ESI): *m*/*z* = 252.10298 $[M+H]^+$ (calcd. 252.10301 for $C_{11}H_{12}O_5NLi_2$)

Dilithium (±)-*erythro*-3-(benzyloxy)aspartate (*erythro*-1a)

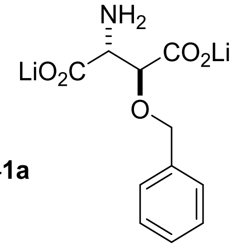

(±)-*erythro*-**1a**

A mixture of (±)-*threo*- and *erythro*-**8a** with 35/65 ratio (17.0 mg, 6.4×10^{-2} mmol) in a solution of LiOH (9.3 mg, 3.9×10^{-1} mmol) in D_2O (0.9 mL) was converted quantitatively into a mixture of (±)-*threo*- and *erythro*-**1a** with 35/65 ratio. (±)-*Erythro*: ^{1}H NMR (500 MHz, D_2O, 25°C) δ 7.44 – 7.35 (m, 5H), 4.67 (d, J = 11.8 Hz, 1H), 4.41 (d, J = 11.8 Hz, 1H), 3.88 (d, J = 6.4 Hz, 1H), 3.43 (d, J = 6.4 Hz, 1H), signal of MeOH at δ 3.34; (±)-*erythro*: ^{13}C NMR (125 MHz, D_2O, 25°C) δ 179.75, 178.27, 138.14, 129.18, 129.03, 129.18, 129.03, 128.73, 84.06 (m), 72.40 (m), 59.43 (m), signal of MeOH: δ 49.50; HRMS (ESI): m/z = 252.10303 $[M+H]^+$ (calcd. 252.10301 for $C_{11}H_{12}O_5NLi_2$)

Dilithium (±)-*threo*-3-(3-fluorobenzyloxy)aspartate (*threo*-1c)

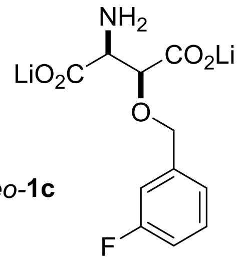

(±)-*threo*-**1c**

(±)-*Threo*-**8c** (40.0 mg, 1.4×10^{-1} mmol) in a solution of LiOH (18.0 mg, 7.5×10^{-1} mmol) in D_2O (0.9 mL) was converted quantitatively into (±)-*threo*-**1c**. ^{1}H NMR (500 MHz, D_2O, 25°C) δ 7.41 – 7.37 (m, 1H), 7.19 – 7.13 (m, 2H), 7.10 – 7.06 (m, 1H), 4.68 (d, *J* = 12.2 Hz, 1H, signal partly overlapping with signal of residual HDO), 4.41 (d, *J* = 12.2 Hz, 1H), 4.19 (d, *J* = 2.3 Hz, 1H), 3.57 (d, *J* = 2.3 Hz, 1H), signal of MeOH at δ 3.34; ^{13}C NMR (125 MHz, D_2O, 25°C) δ 180.26, 178.94, 163.45 (d, *J* = 243.0 Hz), 141.28 (d, *J* = 7.5 Hz), 131.00 (d, *J* = 8.5 Hz), 124.73, 115.60 (d, *J* = 23.5 Hz), 115.42 (d, *J* = 21.6 Hz), 82.80 (m), 72.43 (m), 59.29 (m), signal of MeOH: δ 49.50; HRMS (ESI): *m*/*z* = 270.09350 [M+H]$^+$ (calcd. 270.09359 for $C_{11}H_{11}O_5NFLi_2$)

Dilithium (±)-*threo*-3-(3-trifluoromethylbenzyloxy)aspartate (*threo*-1f)

(±)-*threo*-**1f**

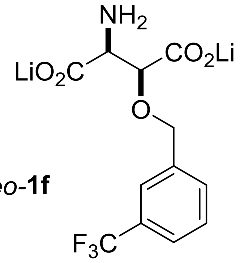

(±)-*Threo*-**8f** (36.0 mg, 1.1×10^{-1} mmol) in a solution of LiOH (15.0 mg, 6.3×10^{-1} mmol) in D_2O (0.75 mL) was converted quantitatively into (±)-*threo*-**1f**. ^{1}H NMR (500 MHz, D_2O, 25°C) δ 7.71 (s, 1H), 7.66 (d, *J* = 7.1 Hz, 1H), 7.63 (d, *J* = 7.9 Hz, 1H), 7.56 (dd, *J* = 7.9, 7.1 Hz, 1H), 4.74 (d, *J* = 12.0 Hz, 1H), 4.48 (d, *J* = 12.0 Hz, 1H), 4.21 (s, 1H), 3.58 (s, 1H), signal of MeOH at δ 3.34; ^{13}C NMR (125 MHz, D_2O, 25°C) δ 179.65, 178.33, 139.07, 132.10 (m), 130.10 (q, *J* = 32.0 Hz), 129.41 (m), 124.99 (q, *J* = 3.3 Hz), 124.95 (q, *J* = 3.3 Hz), 124.56 (q, *J* = 271.9 Hz),

82.35 (m), 71.80 (m), 58.71 (m), signal for MeOH: δ 49.50; HRMS (ESI): *m*/*z* = 320.09025 $[M+H]^+$ (calcd. 320.09039 for $C_{12}H_{11}O_5NF_3Li_2$)

Dilithium (±)-*threo*-3-(3-methylbenzyloxy)aspartate (*threo*-1i)

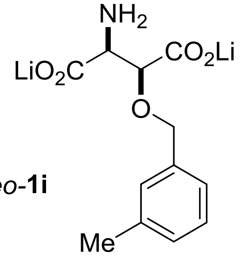

(±)-*threo*-**1i**

(±)-*Threo*-**8i** (35.0 mg, 1.2×10^{-1} mmol) in a solution of LiOH (18.0 mg, 7.5×10^{-1} mmol) in D_2O (0.8 mL) was converted quantitatively into (±)-*threo*-**1i**. ^{1}H NMR (500 MHz, D_2O, 25°C) δ 7.31 (dd, *J* = 7.5, 7.5 Hz, 1H), 7.23 (s, 1H), 7.20 (d, *J* = 7.5 Hz, 1H), 7.19 (d, *J* = 7.5 Hz, 1H), 4.64 (d, *J* = 11.7 Hz, 1H), 4.36 (d, *J* = 11.7 Hz, 1H), 4.19 (d, *J* = 2.6 Hz, 1H), 3.56 (d, *J* = 2.6 Hz, 1H), 2.34 (s, 3H), signal of MeOH at δ 3.34; ^{13}C NMR (125 MHz, D_2O, 25°C) δ 180.06, 178.89, 139.25, 138.35, 129.54 (m), 129.16, 129.14, 125.95, 82.37 (m), 72.74 (m), 59.08 (m), 21.07 (m), signal of MeOH: δ 49.50; HRMS (ESI): *m*/*z* = 266.11860 $[M+H]^+$ (calcd. 266.11866 for $C_{12}H_{14}O_5NLi_2$)

Dilithium (±)-*erythro*-3-(3-methylbenzyloxy)aspartate (*erythro*-1i)

(±)-*erythro*-**1i**

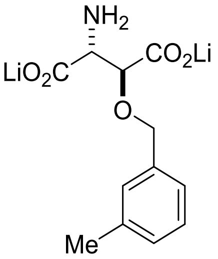

A mixture of (±)-*threo*- and *erythro*-**8i** with 35/65 ratio (15.0 mg, 5.3×10^{-2} mmol) in a solution of LiOH (7.8 mg, 3.2×10^{-1} mmol) in D_2O (0.8 mL) was converted quantitatively into a mixture of (±)-*threo*- and *erythro*-**1i** with 35/65 ratio. (±)-*Erythro*: ^{1}H NMR (500 MHz, D_2O, 25°C) δ 7.32 (dd, *J* = 7.5, 7.4 Hz, 1H), 7.26 (s, 1H), 7.23 (d, *J* = 7.4 Hz, 1H), 7.22 (d, *J* = 7.5 Hz, 1H), 4.64 (d, *J* = 11.8 Hz, 1H), 4.37 (d, *J* = 11.8 Hz, 1H), 3.88 (d, *J* = 6.3 Hz, 1H), 3.43 (d, *J* = 6.3 Hz, 1H), 2.34 (s, 3H), signal of MeOH at δ 3.34; (±)-*erythro*: ^{13}C NMR (125 MHz, D_2O, 25°C) δ 179.71, 178.25, 139.34, 138.20, 129.77, 129.27, 129.21, 126.17, 83.88 (m), 72.36 (m), 60.76 (m), 21.05 (m), signal of MeOH: δ 49.50; HRMS (ESI): *m*/*z* = 266.11858 $[M+H]^+$ (calcd. 266.11866 for $C_{12}H_{14}O_5NLi_2$)

Identification of compounds 7, 8, and 1

Adducts **7a**,**c**,**f**,**i**, **8a**,**c**,**f**,**i**, and dilithium salts **1a**,**c**,**f**,**i** were fully characterized by ^{1}H, ^{13}C NMR, and high resolution mass spectroscopy (vide supra) as, to the best of our knowledge, these compounds have not been reported in the literature so far. Furthermore, the ^{1}H NMR data of chemically synthesized racemic dilithium salt **1a** (R = H) were compared with the ^{1}H NMR data of the dilithium salt of *threo*-**1a** (R = H) that was obtained by treating enzymatically (MAL)

obtained bisammonium salt of *threo*-**1a** (R = H) with LiOH (Figure S1). As the ^{1}H NMR data of both portions of dilithium salts of **1a** exactly matched, we concluded that the chemical synthesis route as depicted in Scheme S1 and the enzymatic synthesis (MAL) route as visualized in Scheme 1 (main text) yield identical products, namely the *threo*-diastereomer of **1a**. The *threo*-configuration of **1a** (R = H) obtained by chemical and enzymatic synthesis (MAL) was established unambiguously by comparison of ^{1}H NMR and HPLC (Figure S3) data with an authentic sample of *threo*-**1a** (R = H) kindly provided by Dr. K. Shimamoto.

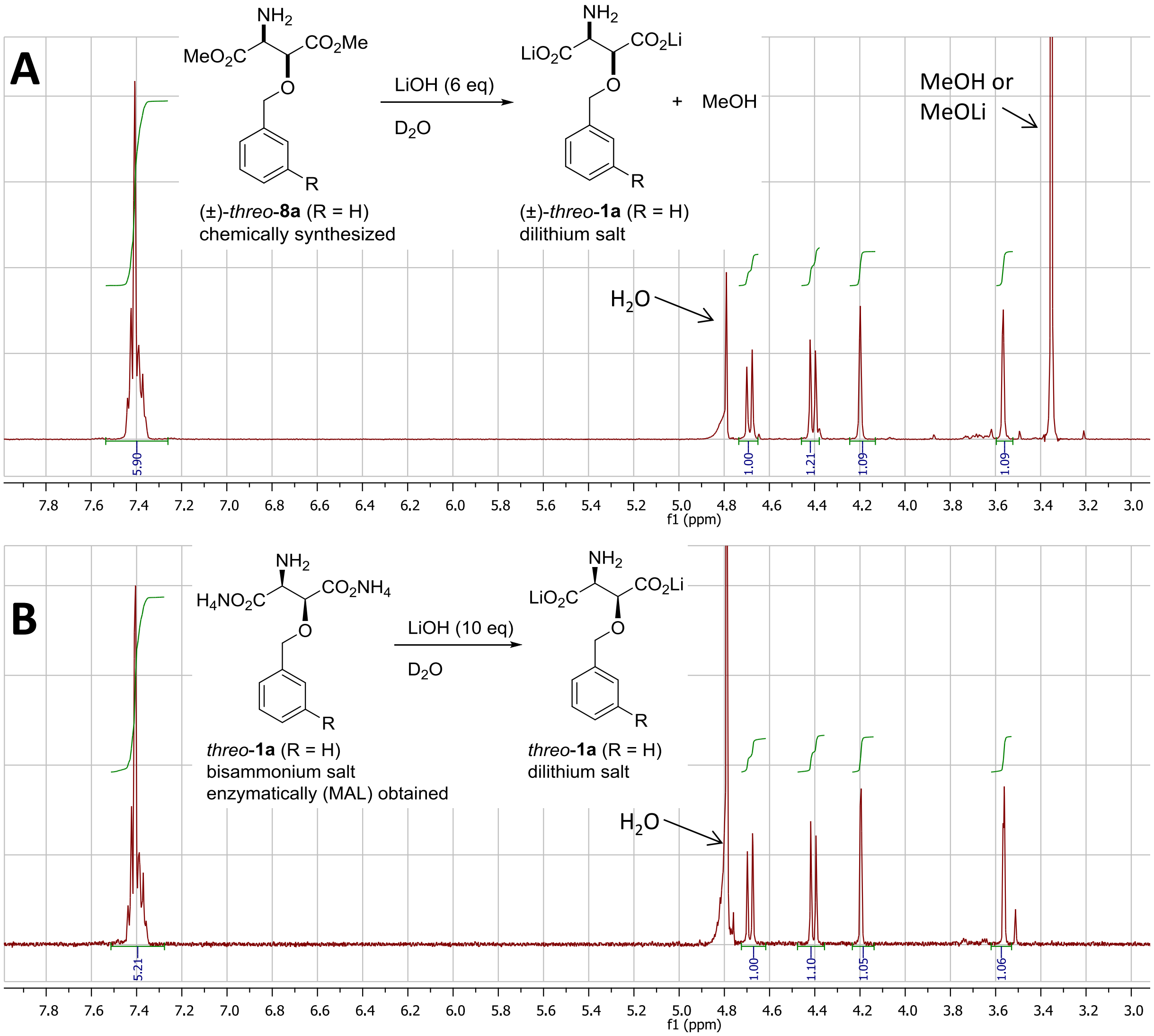

Figure S1. Comparison of ^{1}H NMR data of chemically synthesized racemic dilithium salt **1a** (R = H) (spectrum A) with the ^{1}H NMR data of the dilithium salt of *threo*-**1a** that was obtained by treating enzymatically (MAL) obtained bisammonium salt of *threo*-**1a** (R = H) with LiOH (spectrum B).

Determination of diastereomeric purity of enzymatically (MAL) obtained 1

As described above, we ascertained that the *threo*-isomer of **1a** (R = H) is obtained by chemical as well as by enzymatic synthesis (MAL). We witnessed one set of absorptions in the ^{1}H NMR spectra as depicted in Figure S1 which could be assigned completely to the *threo*-isomer of **1a** (R = H). In theory however, a mixture of *threo*- and *erythro*-isomers of **1a** (R = H) could have been obtained by chemical and/or enzymatic synthesis (MAL) in case the ^{1}H NMR absorptions of the *threo*- and *erythro*-isomers of **1a** exactly overlap. As, to the best of our knowledge, ^{1}H NMR data of the *erythro*-isomer of **1a**, and of any of its derivatives **1b-f**,**h**,**i**, are not available in the literature, we set out to chemically synthesize the *erythro*-isomer of **1a** (R = H) as described in the paragraph *Synthesis of racemic 1* (Scheme S1). The rationale behind this endeavor is to compare the ^{1}H NMR data of the *erythro*-isomer of **1a** (R = H) with the ^{1}H NMR data presented in Figure S1 of which we know unambiguously they represent the *threo*-isomer of **1a**.

As described in the paragraph *Synthesis of racemic 1*, we were able to chemically synthesize the *erythro*-isomer of **1a** (R = H) as a mixture with the *threo*-isomer of **1a** (R = H) in a ~35/65 ratio (*threo*-**1a**/*erythro*-**1a** ~35/65). A ^{1}H NMR spectrum was recorded from this mixture of which the most indicative region (4.75 – 3.40 ppm) is depicted in Figure S2 (spectrum A). Clearly, two sets of aliphatic absorptions are witnessed of which one exactly matches with the absorptions of the chemically and enzymatically (MAL) synthesized *threo*-isomer of **1a** (R = H) (spectrum B) as indicated with the dotted lines connecting spectra A and B. The other set of signals seen in

spectrum A, marked with black triangles, belongs to the *erythro*-isomer of **1a**. Especially the differences in ppm values between the H_2 and H_3 protons of *threo*- and *erythro*-**1a** are significant (H_2: 3.56 *versus* 3.43 ppm; H_3: 4.20 *versus* 3.88 ppm). Differences in ppm values between the benzylic protons of *threo*- and *erythro*-**1a** are small but distinct.

We were also able to chemically synthesize the *erythro*-isomer of *m*-methyl derivative **1i** (R = Me) as a mixture with the *threo*-isomer of **1i** (R = Me) in a ~35/65 ratio (*threo*-**1i**/*erythro*-**1i** ~35/65). Comparison of the ^{1}H NMR spectrum obtained from this mixture with the ^{1}H NMR data of chemically and enzymatically (MAL) synthesized **1i** (R = Me) gave a similar picture as just described for the *threo*- and *erythro*-isomers of **1a** (R = H) and revealed the identical clear differences between chemical shifts of the H_2, H_3, and benzylic protons of the *threo*- and *erythro*-isomers of **1i** (R = Me).

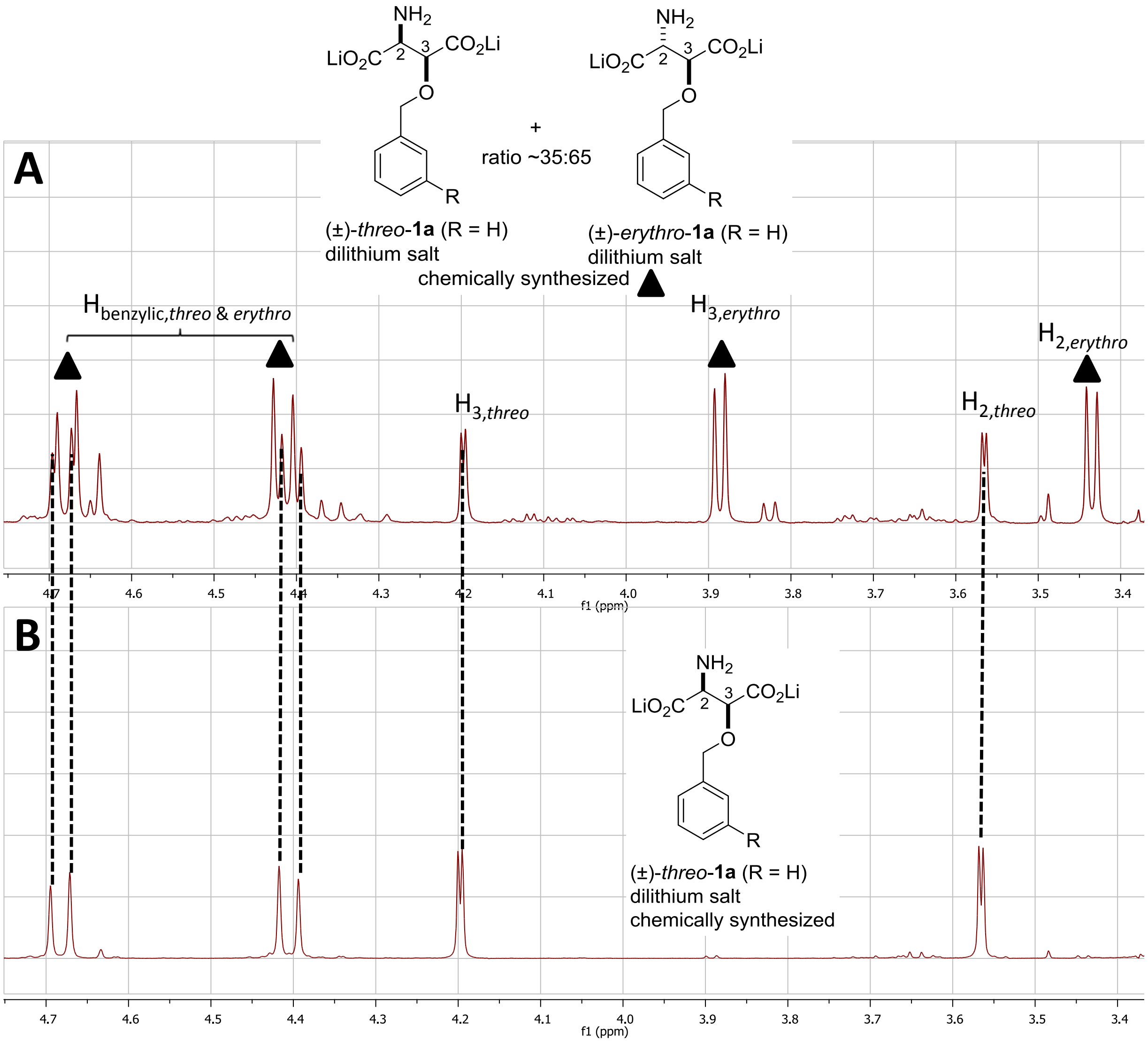

Figure S2. Comparison of a chemically synthesized mixture of (±)-*threo*- and (±)-*erythro*-isomers of **1a** (ratio ~35/65) (spectrum A) and chemically synthesized (±)-*threo*-**1a** (*threo/erythro* ~99/1) (spectrum B).

We conclude that the chemical and enzymatic (MAL) synthesis of **1a,i** (R = H, Me) as depicted in Scheme S1 and Scheme 1 (main text) selectively give access to the *threo*-isomers (diastereomeric excess = ≥ 99%) based on the following observations:

1) the *threo*- and *erythro*-isomers of **1a** (R = H) and of **1i** (R = Me), as obtained from a mixture of *threo*- and *erythro*-isomers of **7a** (R = H) and of **7i** (R = Me) respectively (Scheme S1), can be clearly distinguished by ^{1}H NMR spectroscopy.
2) the ^{1}H NMR data of chemically synthesized **1a**,**i** (R = H, Me), obtained from pure (±)-*threo*-**7a**,**i** (Scheme S1), and the ^{1}H NMR data of enzymatically (MAL) synthesized **1a**,**i** (R = H, Me) exclusively show the signals belonging to the *threo*-isomers of **1a**,**i** (R = H, Me).

We assume that all other chemically obtained derivatives of **1** (i.e. **1c**,**f**) and enzymatically (MAL) obtained derivatives of **1** (i.e. **1b-f**,**h**) were also exclusively obtained with *threo*-configurations (diastereomeric excess = ≥99%). We base this assumption on the following facts:

1) for each enzymatically (MAL) obtained derivative **1a-f**,**h**,**i** only one set of aliphatic ^{1}H NMR signals is observed.
2) these sets of aliphatic ^{1}H NMR signals of enzymatically (MAL) obtained derivatives **1a-f**,**h**,**i** are all identical to each other (ppm values, coupling constants)
3) the set of aliphatic ^{1}H NMR signals of chemically synthesized *threo*-isomer **1a** exactly matches the set of aliphatic ^{1}H NMR signals of enzymatically (MAL) obtained *threo*-isomer **1a**, and as well exactly matches the set of aliphatic ^{1}H NMR signals of an authentic sample of *threo*-isomer **1a**.
4) *threo* and *erythro*-isomers of two chemically synthesized derivatives of **1** (**1a**: R = H and **1i**: R = Me) are clearly distinguished by ^{1}H NMR spectroscopy on basis of two different sets of aliphatic signals.

5) the sets of aliphatic ^{1}H NMR signals of chemically synthesized *threo*-isomers of **1a**,**c**,**f**,**i** are all similar to each other (ppm values, coupling constants).
6) the sets of aliphatic ^{1}H NMR signals of chemically synthesized *erythro*-isomers **1a** and **1i** are similar to each other (ppm values, coupling constants).

Enantiomeric excess determination of enzymatically (MAL) obtained 1

Chemically synthesized racemic lithium salts of *threo*-**1a**,**c**,**f**,**i** (Scheme S1) served as reference compounds to determine the enantiomeric excess of enzymatically obtained *threo*-**1a**,**c**,**f**,**i** with chiral phase HPLC. HPLC conditions were as follows: Nucleosil chiral-1 column with 0.5 mM aq. $CuSO_4$ as mobile phase and a flow rate of 1.0 mL/min at 60°C (UV detection at 210 nm).

Results revealed that enzymatically obtained *threo*-**1a**,**c**,**f**,**i** were all obtained with ≥99% enantiomeric excess (see Figures S3, S5-S7 and Table S1). We assume that the other four enzymatically (MAL) obtained derivatives *threo*-**1b**,**d**,**e**,**h** were also obtained with ≥99% enantiomeric excess. We base this assumption on the following facts:

1) the four enzymatically obtained derivatives *threo*-**1b**,**d**,**e**,**h** only show one peak under above-mentioned HPLC conditions (like enantiopure derivatives *threo*-**1a**,**c**,**f**,**i**) (see Figure S8)

2) optical rotations of *threo*-**1b**,**d**,**e**,**h**, in terms of signs *and* values, are identical to enantiopure derivatives *threo*-**1a**,**c**,**f**,**i** (vide infra, Table S1).

Absolute configuration determination of enzymatically (MAL) obtained 1a-f,h,i

The absolute configuration of enzymatically (MAL) obtained enantiopure *threo*-**1a** was determined by chiral phase HPLC and by optical rotation (Table S1). As described in the

previous paragraph, the enantiomeric excess of enzymatically obtained *threo*-**1a** was determined to be ≥99% by chiral phase HPLC. The absolute configuration of *threo*-**1a** was assigned to be (2*S*,3*S*) unambiguously by comparison with HPLC data of an authentic sample of 2*S*,3*S*-(−)-*threo*-benzyloxyaspartate **1a** (L-TBOA) kindly provided by Dr. K. Shimamoto. Moreover, a negative optical rotation of enzymatically (MAL) obtained *threo*-**1a** was found which is in line with the negative rotation reported by Shimamoto et al. for 2*S*,3*S*-(−)-*threo*-benzyloxyaspartate **1a** (L-TBOA) (see Table S1).[1]

The absolute configurations of the major enantiomers of **1b-f**,**h**,**i** are assumed to be 2*S*,3*S* as well on basis of their identical rotations, in terms of sign *and* value, as compared to **1a** of which the 2*S*,3*S*-configuration was established unambiguously (see Table S1).

Table S1. Absolute configuration determination of enzymatically (MAL) obtained enantiopure *threo*-**1a** and tentative assignment of the absolute configuration of *threo*-**1b-f**,**h**,**i**.

Product	R^1	R^2	R^3	e.e. (%) (HPLC)	Optical rotation ($[\alpha]^{25}_{D}$) (1N HCl (aq)/DMSO 1/1)	Absolute configuration of major enantiomer
**1a**	H	H	H	≥99	−13.6° (c 0.35)[e]	(2*S*,3*S*)[c]
**1b**	F	H	H	≥99[a]	−12.4° (c 0.35)	(2*S*,3*S*)[d]
**1c**	H	F	H	≥99	−12.2° (c 0.35)	(2*S*,3*S*)[d]
**1d**	H	H	F	≥99[a]	−12.6° (c 0.35)	(2*S*,3*S*)[d]
**1e**	CF_3	H	H	≥99[a]	−12.4° (c 0.35)	(2*S*,3*S*)[d]
**1f**	H	CF_3	H	≥99	−9.2° (c 0.30)	(2*S*,3*S*)[d]
**1g**	H	H	CF_3	n.a.[b]	n.a.	n.a.
**1h**	Me	H	H	≥99[a]	−13.7° (c 0.35)	(2*S*,3*S*)[d]
**1i**	H	Me	H	≥99	−12.8° (c 0.35)	(2*S*,3*S*)[d]
**1j**	H	H	Me	n.a.	n.a.	n.a.

[a] Enantiomeric excess is assumed to be ≥99% because enzymatically obtained *threo*-**1b**,**d**,**e**,**h** only show one peak in HPLC chromatogram (like enantiopure *threo*-**1a**,**c**,**f**,**i**) (see Figure S8) and because optical rotations of *threo*-**1b**,**d**,**e**,**h**, in terms of signs *and* values, are identical to enantiopure *threo*-**1a**,**c**,**f**,**i**.
[b] n.a. = not available (i.e. compounds **1g**,**j** could not be obtained with MAL).
[c] Absolute configuration determined unambiguously by comparison with authentic sample of L-TBOA (i.e. 2*S*,3*S*-(−)-TBOA).
[d] The absolute configurations of the major enantiomers of **1b-f**,**h**,**i** are assumed on basis of the identical rotations of **1b-f**,**h**,**i**, in terms of sign *and* value, as compared to **1a** of which the 2*S*,3*S*-configuration was established unambiguously.
[e] Lit: $[\alpha]^{25}_{D}$ = −473.6° (c 0.68 1N HCl)[6]

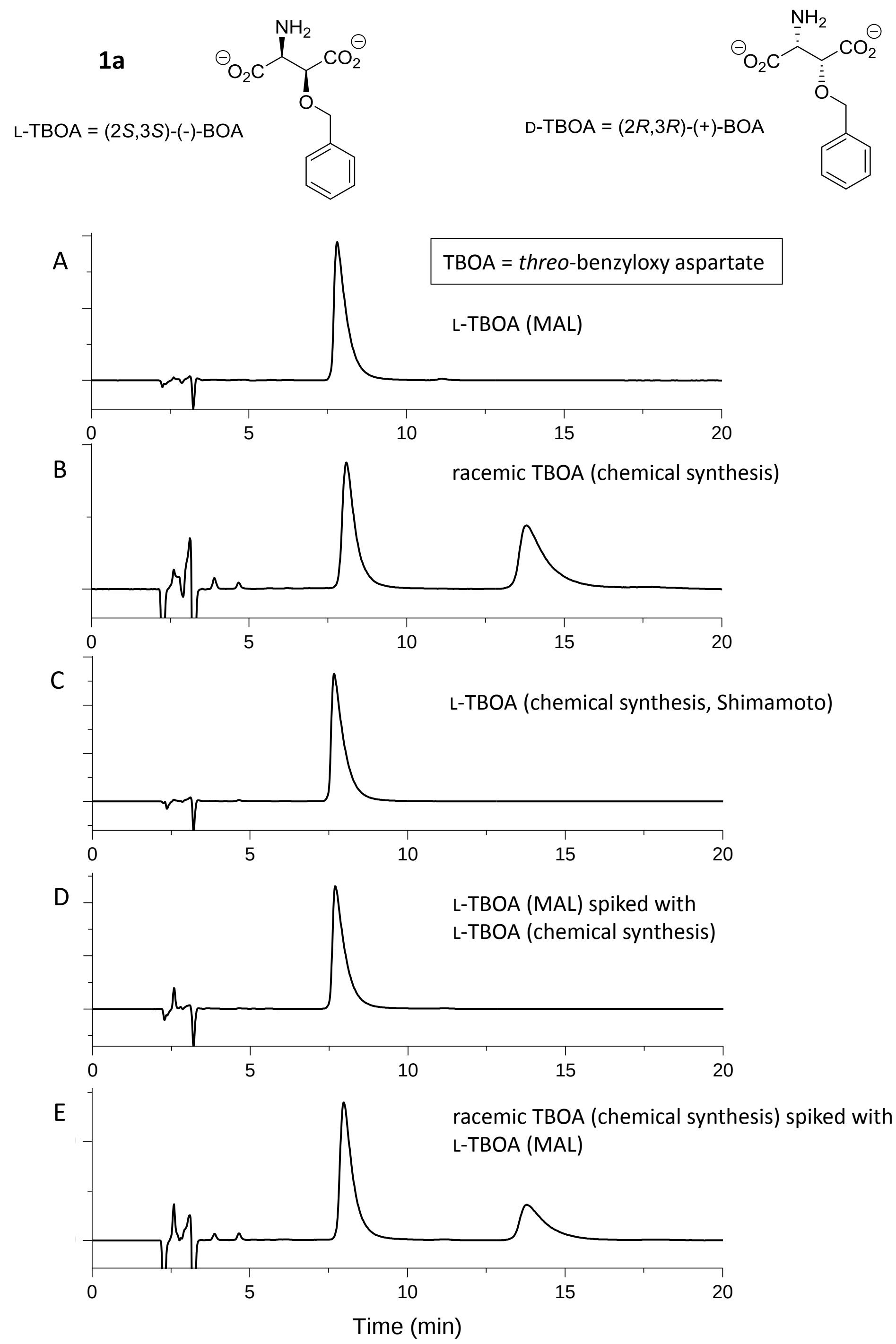

Figure S3. Determination of the enantiomeric excess and absolute configuration of product **1a** (TBOA) of the MAL(L384G)-catalyzed amination of 2-benzyloxyfumarate (**2a**) by using HPLC with a chiral stationary phase. A) Chromatogram of enzymatically (MAL) prepared **1a**. B) Chromatogram of chemically synthesized (±)-*threo*-**1a** (i.e. racemic). C) Chromatogram of authentic sample of chemically synthesized 2*S*,3*S*-(−)-**1a** (L-TBOA) (kindly provided by Dr. K. Shimamoto). D) Chromatogram of a mixture of enzymatically (MAL) prepared **1a** and chemically synthesized 2*S*,3*S*-(−)-**1a**. E) Chromatogram of a mixture of enzymatically (MAL) prepared **1a** spiked with chemically synthesized (±)-**1a**. This analysis showed that enzymatically (MAL) prepared **1a** has the L configuration and was obtained with an enantiomeric excess of ≥99%.

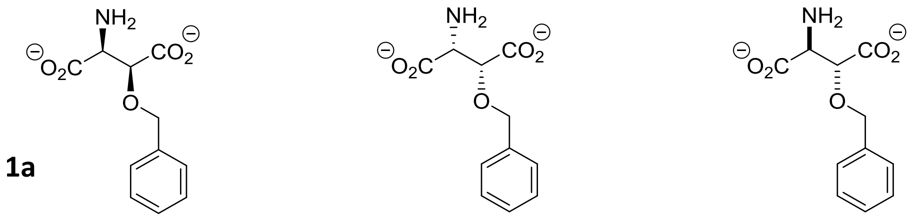

L-TBOA = (2*S*,3*S*)-(-)-BOA D-TBOA = (2*R*,3*R*)-(+)-BOA (±)-EBOA

TBOA = *threo*-benzyloxy aspartate
EBOA = *erythro*-benzyloxy aspartate

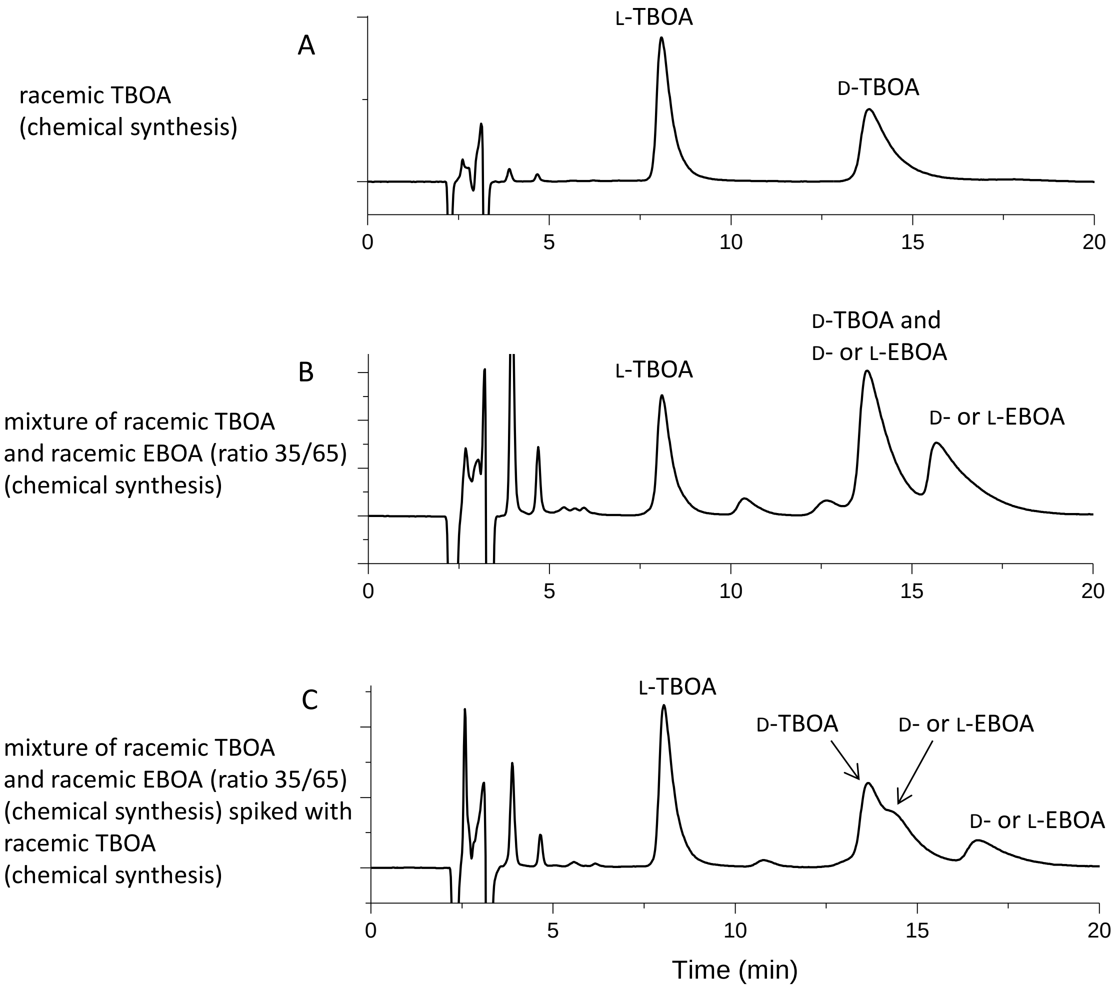

Figure S4. A) Chromatogram of chemically synthesized (±)-*threo*-**1a** (i.e. racemic). B) Chromatogram of a mixture of chemically synthesized (±)-*threo*-**1a** (i.e. racemic) and (±)-*erythro*-**1a** (i.e. racemic) with ratio 35/65. C) Chromatogram of a mixture of chemically synthesized (±)-*threo*-**1a** (i.e. racemic) and (±)-*erythro*-**1a** (i.e. racemic) with ratio 35/65 spiked with chemically synthesized (±)-*threo*-**1a** (i.e. racemic).

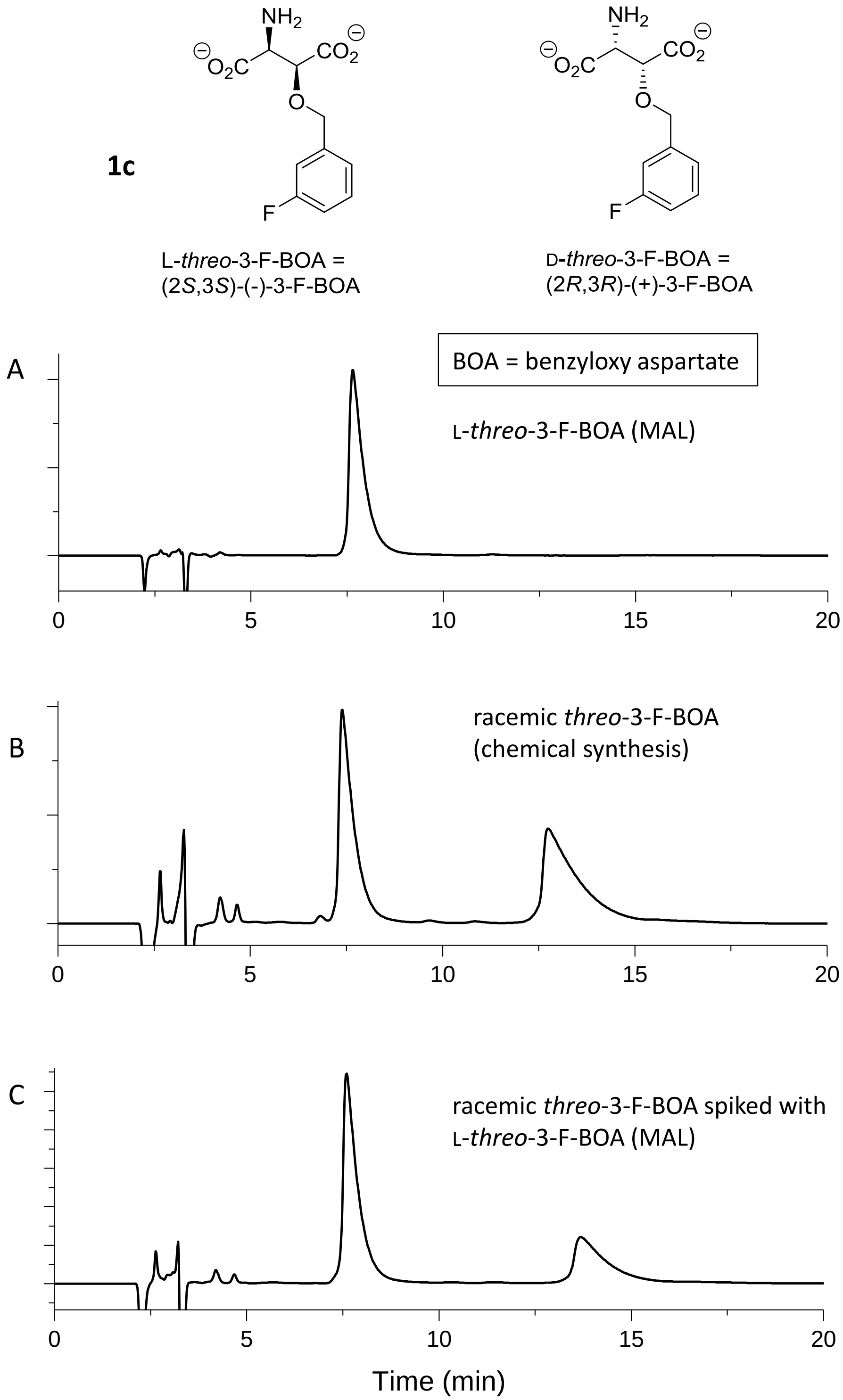

Figure S5. Determination of the enantiomeric excess of product **1c** of the MAL(L384G)-catalyzed amination of 2-benzyloxyfumarate (**2c**) by using HPLC with a chiral stationary phase. A) Chromatogram of enzymatically (MAL) prepared **1c**. B) Chromatogram of chemically synthesized (±)-*threo*-**1c** (i.e. racemic). C) Chromatogram of enzymatically (MAL) prepared **1c** spiked with chemically synthesized (±)-**1c**. This analysis showed that enzymatically (MAL) prepared **1c** was obtained with an enantiomeric excess of ≥99%.

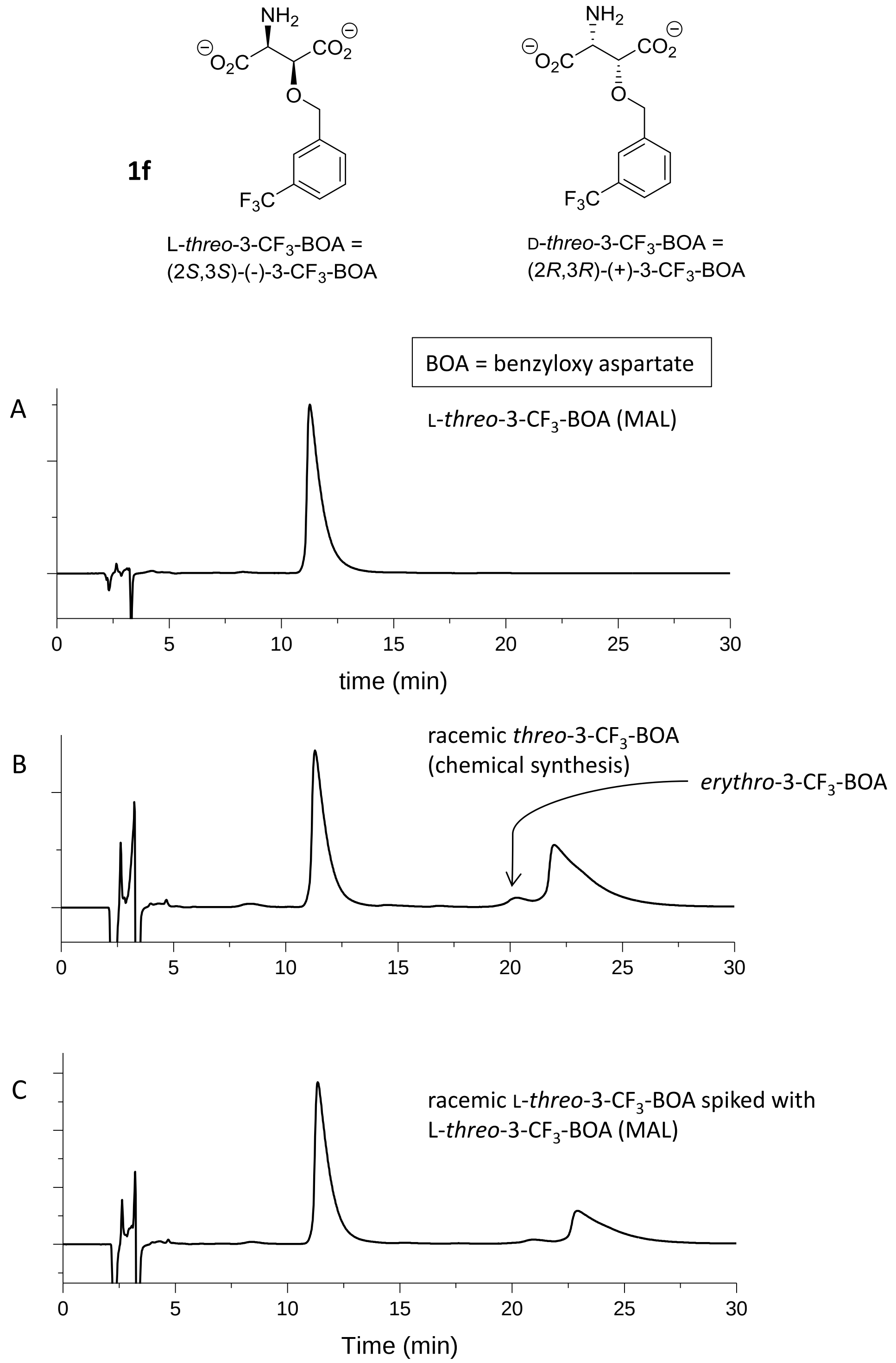

Figure S6. Determination of the enantiomeric excess of product **1f** of the MAL(L384G)-catalyzed amination of 2-benzyloxyfumarate (**2f**) by using HPLC with a chiral stationary phase. A) Chromatogram of enzymatically (MAL) prepared **1f**. B) Chromatogram of chemically synthesized (±)-*threo*-**1f** (i.e. racemic). C) Chromatogram of enzymatically (MAL) prepared **1f** spiked with chemically synthesized (±)-**1f**. This analysis showed that enzymatically (MAL) prepared **1f** was obtained with an enantiomeric excess of ≥99%.

1i

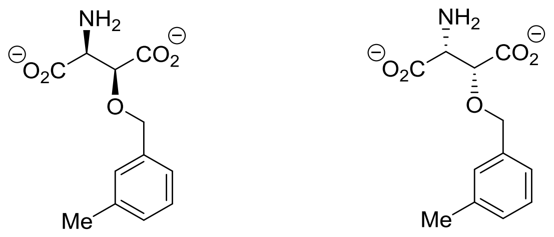

L-*threo*-3-Me-BOA = (2*S*,3*S*)-(-)-3-Me-BOA

D-*threo*-3-Me-BOA = (2*R*,3*R*)-(+)-3-Me-BOA

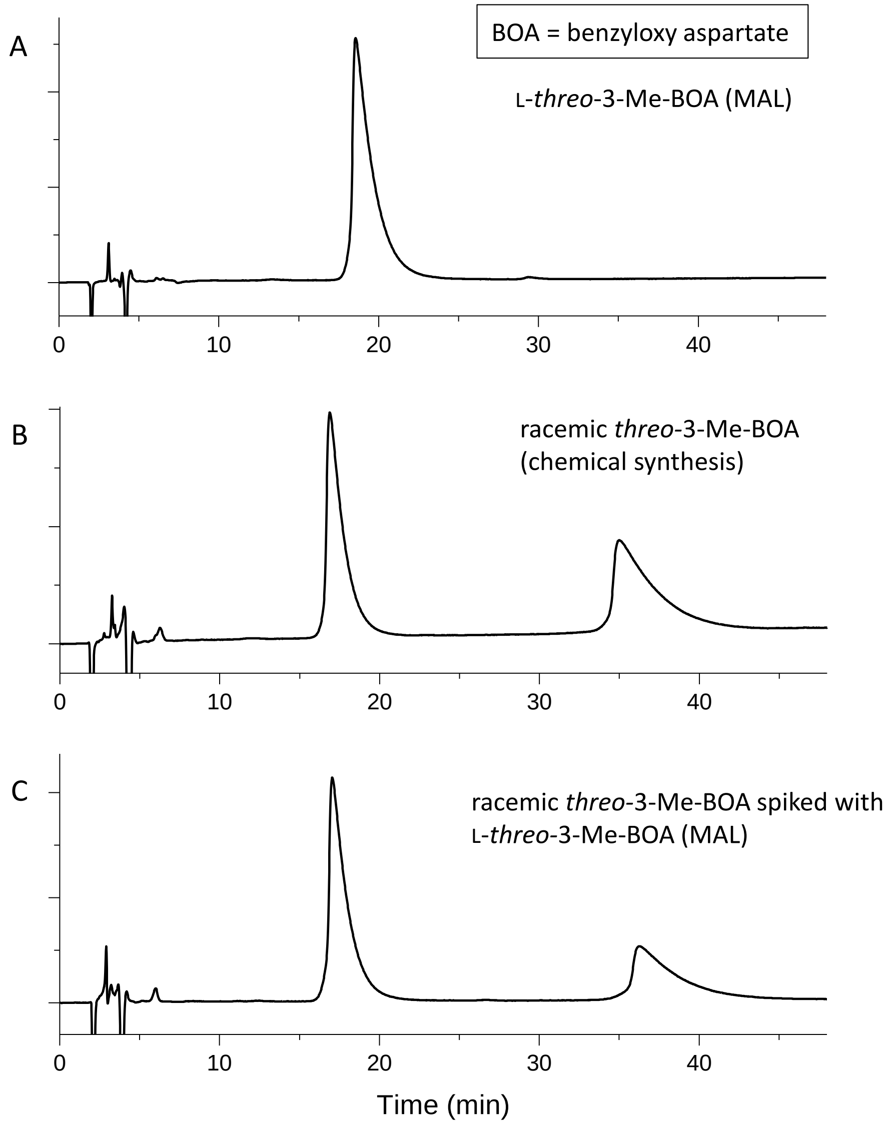

Figure S7. Determination of the enantiomeric excess of product **1i** of the MAL(L384G)-catalyzed amination of 2-benzyloxyfumarate (**2i**) by using HPLC with a chiral stationary phase. A) Chromatogram of enzymatically (MAL) prepared **1i**. B) Chromatogram of chemically synthesized (±)-*threo*-**1i** (i.e. racemic). C) Chromatogram of enzymatically (MAL) prepared **1i** spiked with chemically synthesized (±)-**1i**. This analysis showed that enzymatically (MAL) prepared **1i** was obtained with an enantiomeric excess of ≥99%.

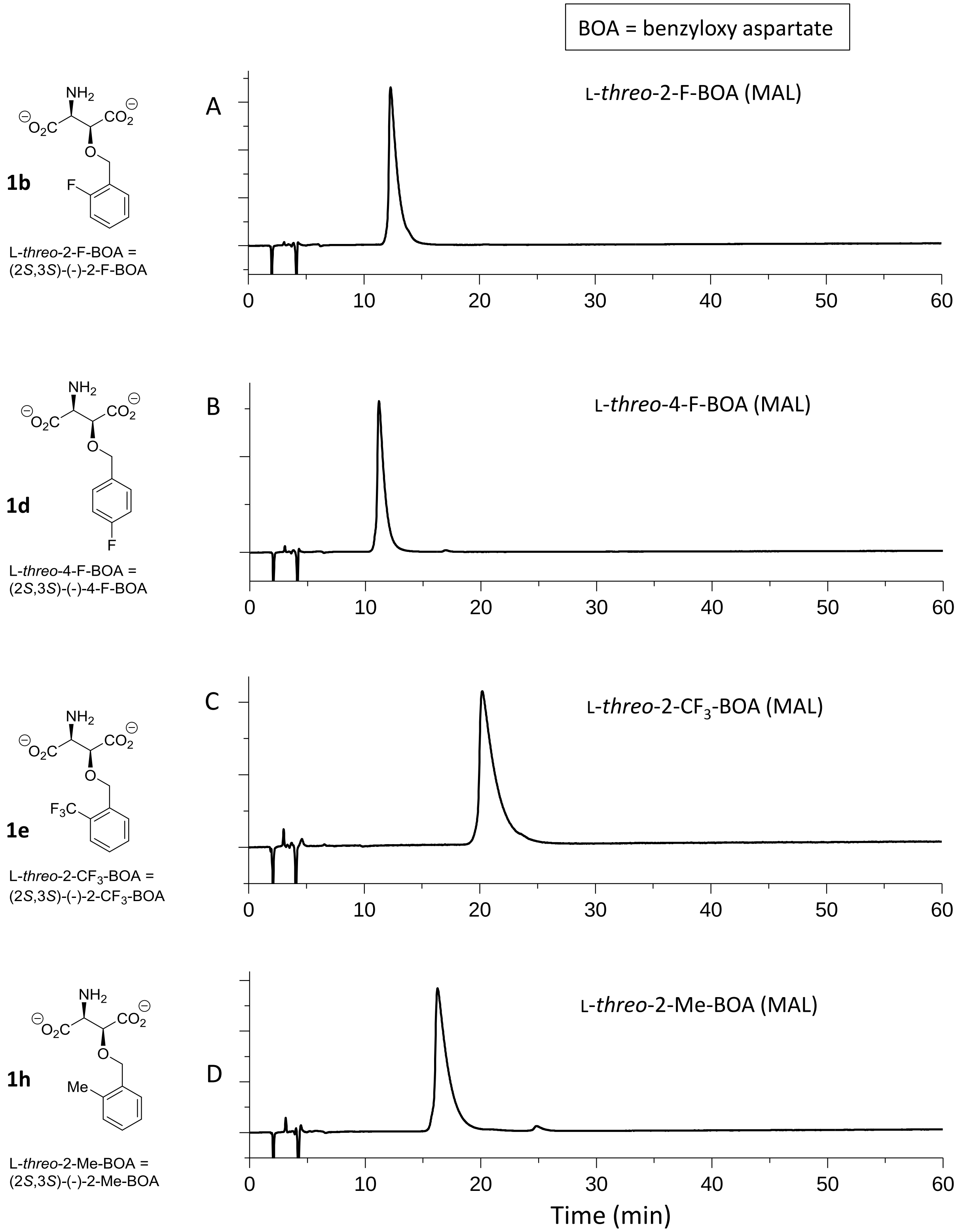

Figure S8. HPLC chromatograms of A) enzymatically (MAL) prepared **1b**; B) enzymatically (MAL) prepared **1d**; C) enzymatically (MAL) prepared **1e**; D) enzymatically (MAL) prepared **1h**.

Kinetic resolution of DL-TBOA

The racemic mixture of DL-TBOA (5 mg, 0.021 mmol) was dissolved in 50 mM Tris buffer, pH 8.0 (5 mL), containing $MgCl_2$ (20 mM) and KCl (1 mM). The reaction was started by the addition of freshly purified L384A enzyme (0.1 mol%) and the reaction mixture was incubated at room temperature. At specific time points (3 h, 6 h and 24 h) an aliquot (1 mL) was removed and the reaction stopped by incubating the sample at 100°C for 10 min. The precipitated enzyme was removed by centrifugation (13300 rpm for 10 min). The supernatant was filtered (0.2 µm filter cartridge) followed by injection on chiral HPLC (Nucleosil chiral-1 column) using 0.5 mM aq. $CuSO_4$ as mobile phase and a flow rate of 1.0 mL/min at 60°C (UV detection at 254 nm).

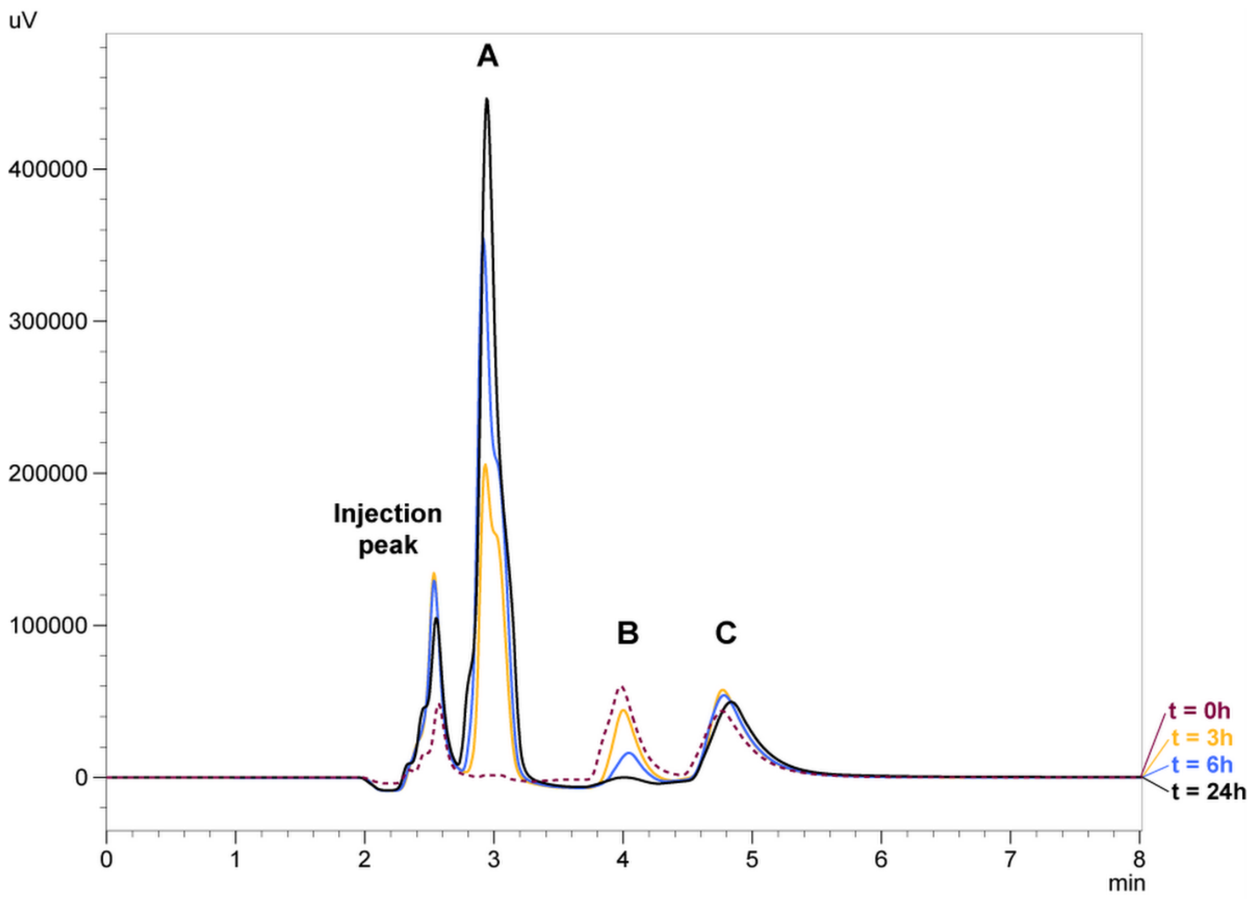

Figure S9. HPLC chromatograms illustrating the kinetic resolution of DL-TBOA by using MAL(L384A) as biocatalyst. **A:** The formation of 2-benzyloxyfumarate over time. **B:** The depletion of L-TBOA over time. **C:** The concentration of D-TBOA stays constant over time.

References

(1) Fan, M. J., Li, G. Q., Liang, Y. M., *Tetrahedron* **2006**, *62*, 6782.

(2) Raj, H., Szymanski, W., de Villiers, J., Rozeboom, H. J., Puthan Veetil, V., Reis, C. R., de Villiers, M., Dekker, F. J., de Wildeman, S., Quax, W. J., Thunnissen, A. M.W.H., Feringa, B. L., Janssen, D. B., Poelarends, G. J., *Nat. Chem.* **2012**, *4*, 478.

(3) Raj, H., Weiner, B., Puthan Veetil, V., Reis, C. R., Quax, W. J., Janssen, D. B., Feringa, B. L., Poelarends, G. J., *ChemBioChem* **2009**, *10*, 2236.

(4) Waddell, W. J., *J. Lab. Clin. Med.* **1956**, *48*, 311.

(5) Masruri, A. C. Wills, M. D. McLeod, *J. Org. Chem*. **2011**, *76*, 358.

(6) K. Shimamoto, Y. Shigeri, Y. Yasuda-Kamatani, B. Lebrun, N. Yumoto, T. Nakajima, *Bioorg. Med. Chem. Lett*. **2000**, *10*, 2407.
